# Inhibition of DNA damage response at telomeres improves the detrimental phenotypes of Hutchinson–Gilford Progeria Syndrome

**DOI:** 10.1038/s41467-019-13018-3

**Published:** 2019-11-18

**Authors:** Julio Aguado, Agustin Sola-Carvajal, Valeria Cancila, Gwladys Revêchon, Peh Fern Ong, Corey Winston Jones-Weinert, Emelie Wallén Arzt, Giovanna Lattanzi, Oliver Dreesen, Claudio Tripodo, Francesca Rossiello, Maria Eriksson, Fabrizio d’Adda di Fagagna

**Affiliations:** 1 IFOM Foundation—FIRC Institute of Molecular Oncology Foundation, Via Adamello 16, 20139 Milan, Italy; 2 Australian Institute for Bioengineering and Nanotechnology, The University of Queensland, St. Lucia, QLD 4072 Australia; 3 Department of Biosciences and Nutrition, Center for Innovative Medicine, Karolinska Institutet, Huddinge, Sweden; 4 Tumor Immunology Unit, Department of Health Sciences, University of Palermo, Palermo, Italy; 5 Cell Ageing, Skin Research Institute Singapore, 8A Biomedical Grove, #06-06, Immunos, 138648 Singapore; 6 Istituto di Genetica Molecolare, Consiglio Nazionale delle Ricerche (IGM-CNR), Unit of Bologna, 40126 Bologna, Italy; 7 IRCCS Istituto Ortopedico Rizzoli, 40126 Bologna, Italy; 8 Istituto di Genetica Molecolare, Consiglio Nazionale delle Ricerche (IGM-CNR), Via Abbiategrasso 207, 27100 Pavia, Italy

**Keywords:** Antisense oligonucleotide therapy, Antisense oligonucleotide therapy, Telomeres, Double-strand DNA breaks

## Abstract

Hutchinson–Gilford progeria syndrome (HGPS) is a genetic disorder characterized by premature aging features. Cells from HGPS patients express progerin, a truncated form of Lamin A, which perturbs cellular homeostasis leading to nuclear shape alterations, genome instability, heterochromatin loss, telomere dysfunction and premature entry into cellular senescence. Recently, we reported that telomere dysfunction induces the transcription of telomeric non-coding RNAs (tncRNAs) which control the DNA damage response (DDR) at dysfunctional telomeres. Here we show that progerin-induced telomere dysfunction induces the transcription of tncRNAs. Their functional inhibition by sequence-specific telomeric antisense oligonucleotides (tASOs) prevents full DDR activation and premature cellular senescence in various HGPS cell systems, including HGPS patient fibroblasts. We also show in vivo that tASO treatment significantly enhances skin homeostasis and lifespan in a transgenic HGPS mouse model. In summary, our results demonstrate an important role for telomeric DDR activation in HGPS progeroid detrimental phenotypes in vitro and in vivo.

## Abstract (web summary)

Hutchinson–Gilford progeria syndrome causes premature aging. Here the authors show that activation of the DNA damage response at dysfunctional telomeres and transcription of telomeric non-coding RNAs contributes to the pathogenesis, which can be ameliorated by treatment with sequence-specific telomeric antisense oligonucleotides.

## Introduction

Hutchinson–Gilford Progeria Syndrome (HGPS) is a rare human genetic disease most often caused by heterozygous mutations in the *LMNA* gene, the most common being c.1824C>T, encoding lamin A and lamin C^1,2^. This mutation results in aberrant splicing, which leads to the expression of a truncated form of lamin A protein called progerin. Compared with normal fibroblasts, HGPS fibroblasts exhibit nuclear shape abnormalities, loss of heterochromatin, as indicated by low levels of H3K9me3, H3K27me3, and of heterochromatin protein 1 alpha (HP1α)^3^. Interestingly, progerin expression is sufficient to induce cellular senescence^4^ and its accumulation is known to affect stem cell function both in vitro^5^ and in the skin of HGPS mouse models^6^. Progerin levels accumulate in the skin and arteries of healthy aged individuals and in dermal fibroblasts and terminally differentiated keratinocytes^7–10^.

Importantly, HGPS nuclei accumulate DNA damage and markers of DNA damage response (DDR) activation, and exhibit chromosomal instability proposed to be associated with deficiencies in the DNA double-strand break (DSB) repair^11,12^ and caused by accelerated telomere shortening^13,14^ and dysfunction^15,16^. Telomerase expression in progerin-expressing human cells was found to suppress DDR activation, improve cell proliferation rates, and restore many senescence-associated misregulated genes^17^, suggesting that telomere dysfunction plays a role in HGPS.

Thus, telomere dysfunction and its consequences are emerging as key features in HGPS. The difficulty to therapeutically implement the use of telomerase ectopic expression argues for the development of strategies to control telomere dysfunction. These approaches will allow to both better understand the pathogenesis of the disease and to test potential therapeutic approaches.

At the apex of the DDR-signaling network, following DSB generation the protein kinase ataxia telangiectasia mutated (ATM) is activated and it phosphorylates the histone variant H2AX at serine 139 (named γH2AX)^18,19^. This event is required for the secondary recruitment of DDR factors to the DSB to form the so-called DDR foci, including the autophosphorylated form of ATM (pATM), p53-binding protein 1 (53BP1), and phosphorylated KRAB-associated protein 1 (pKap1).

We recently demonstrated that noncoding RNAs are generated at sites of DNA damage and control DDR activation (reviewed in^20^). Upon DSBs induction, RNA polymerase II is recruited to DSBs in a MRE11/RAD50/NBS1 (MRN)-dependent manner, where it synthesizes damage-induced long noncoding RNAs (dilncRNAs). dilncRNAs are subsequently processed by the endoribonucleases DROSHA and DICER into shorter noncoding RNAs termed DNA damage response RNAs (DDRNAs), which support a full DDR activation and secondary recruitment of DDR factors^21–24^.

We have also shown that telomere dysfunction, just like DSBs, induces the transcription of telomeric dilncRNAs (tdilncRNAs) and telomeric DDRNAs (tDDRNAs) from both DNA strands of the telomere^25,26^. Such transcripts are necessary for DDR activation and maintenance at dysfunctional telomeres. Most importantly, we demonstrated that the use of sequence-specific blocking antisense oligonucleotides (ASOs) inhibits the functions of tDDRNAs and tdilncRNAs and blocks telomere-specific DDR both in cultured cells and in a mouse model bearing uncapped telomeres^25^.

In this study, we demonstrate that progerin-induced telomere dysfunction results in the transcription of tncRNAs, and that their functional inhibition by telomeric sequence-specific antisense oligonucleotides (tASOs) improves tissue homeostasis and extends healthspan and lifespan in a transgenic HGPS mouse model. Hence, our results reveal the contribution of telomeric DDR signaling in HGPS pathogenesis and validate ASO-based strategies as a promising approach to target telomeric dysfunction.

## Results

### Progerin induces tncRNAs and tASO reduces DDR and rescues proliferation

**Fig. 1:**
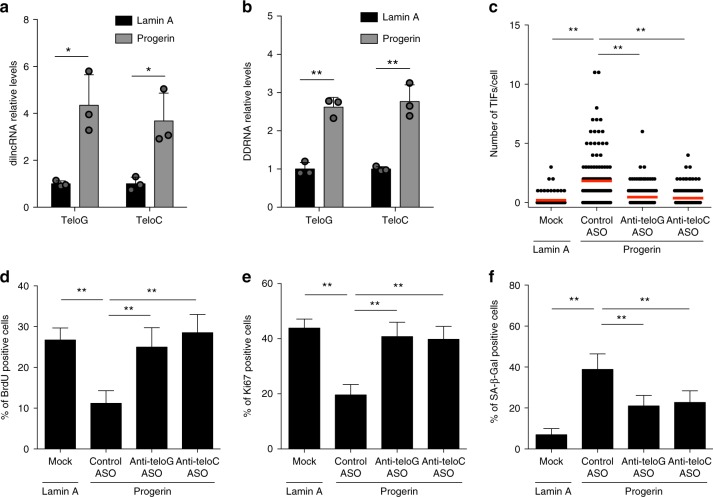
Inhibition of progerin-induced tncRNAs reduces proliferative defects and cellular senescence. **a**, **b** Total cell RNA was purified from human fibroblasts transduced with a retroviral vector expressing either lamin A or progerin. **a** tdilncRNAs were quantified by strand-specific RT-qPCR. Error bars represent s.d., *n* = 3 independent experiments. *P < 0.05; two-tailed Student’s *t* test. **b** tDDRNAs were quantified by miScript PCR amplification of gel-extracted small RNAs (shorter than 40 nucleotides). Error bars represent s.d., *n* = 3 independent experiments. **P < 0.01; two-tailed Student’s *t* test. **c** Human fibroblasts were transfected with the indicated ASOs and 24 h later transduced with a retroviral vector expressing either lamin A or progerin. Fixed cells were stained for 53BP1 and TRF2 to quantify telomere dysfunction-induced foci (TIFs) as determined by 53BP1 co-localizing with TRF2. *n* = 3 independent experiments. **P < 0.01; one-way ANOVA with multiple-comparison post-hoc corrections. At least 100 cells per sample were analyzed. **d**–**f** Cells from experiments shown in **c** were pulsed with BrdU for 8 h and stained for BrdU (**d**), Ki67 (**e**), and SA-β-Gal activity (**f**). Bar graphs show the percentage of positive cells ± 95% confidence interval. *n* = 3 independent experiments. **P < 0.01; Chi-squared test. Source data are provided as a Source Data file

To explore the potential generation of telomere transcripts and study their role in an amenable human cell model of HGPS, we expressed WT or HGPS mutant form of the *LMNA* gene product (lamin A or progerin, respectively) through retroviral delivery in human skin fibroblasts (Supplementary Fig. 1a). As compared with lamin A-overexpressing and control uninfected cells, progerin expression resulted in increased number of telomere dysfunction-induced foci (TIFs) per cell (Supplementary Fig. 1b, c), a decrease in BrdU incorporation and in the percentage of Ki67-positive cells, two independent measures of cell proliferation (Supplementary Fig. 1d, e). Consistent with the observed increased number of TIFs, progerin expression led to a significant induction of both G-rich (teloG) and C-rich (teloC) strands of tdilncRNAs and tDDRNAs (Fig. 1a, b, respectively). In addition, progerin expression led to a loss of H3K9me3 and HP1α heterochromatin marks and lamin B1 protein levels (Supplementary Fig. 1f) and altered nuclear envelope shape, as determined visually and as measured by reduced nuclear shape circularity (Supplementary Fig. 1g).

We next tested the impact of the direct inhibition of telomeric noncoding RNA (tncRNA) functions by the use of sequence-specific blocking ASOs designed against telomeric repeats (tASOs). We observed that delivery by transfection of tASOs complementary to either telomeric RNA strands (anti-teloG and anti-teloC), but not a control ASO against an unrelated sequence, significantly reduced the number of TIFs in progerin-expressing cells, down to the levels of control cells (Fig. 1c and Supplementary Fig. 1h).

Next, we tested the impact that telomeric DDR inhibition had on cell proliferation. Both anti-teloG and anti-teloC ASOs restored the proliferation rate of progerin-expressing cells to the levels of lamin A-expressing control cells, as independently measured by BrdU incorporation and Ki67 (Fig. 1d, e). Persistent DDR activation at telomeres induces cellular senescence^27–31^. Hence, we tested the impact of telomeric DDR inhibition on cellular senescence by measuring senescence-associated-β-galactosidase (SA-β-gal) activity. We observed that treatments with both tASOs, but not with control ASO, strongly decreased the percentage of progerin-induced SA-β-gal-positive cells (Fig. 1f and Supplementary Fig. 1i). In all these assays, tASO treatments left lamin A and progerin expression levels unaltered (Supplementary Fig. 1j, k) and had no impact on cell proliferation in control lamin A-expressing cells (Supplementary Fig. 1l, m).

Next, we investigated the potential link between telomeric DDR activation and other features previously reported to be altered by progerin expression, namely heterochromatin reduction^17,32^, lamin B1 downregulation^33^, and altered nuclear shape^3^. We observed that lamin B1 levels, as well as the heterochromatin marks H3K9me3 and HP1α, which were reduced in progerin-expressing cells, were left unaltered by telomeric DDR inhibition by tASO treatments (Supplementary Fig. 1n). Similarly, aberrant nuclear shape caused by progerin expression was unaffected (Supplementary Fig. 2a, b).

TERRA are telomeric transcripts whose sequence contains UUAGGG repeats^34,35^ that may be recognized by anti-teloG ASO. TERRA levels, as detected at multiple subtelomeric regions, were not affected upon tASO treatments (Supplementary Fig. 2c) although their levels were mildly increased in progerin-expressing cells, consistent with previous reports of TERRA induction at dysfunctional telomeres^36,37^.

These results demonstrate that ASO-mediated telomere-specific inhibition of DDR signaling at dysfunctional telomeres in progerin-expressing normal human skin fibroblasts is sufficient to prevent their proliferative defects while leaving heterochromatic marks and nuclear shape unaltered. Therefore, telomeric DDR signaling plays an important role in causing proliferative defects and the senescent phenotype in progerin-expressing cells and demonstrates that tASOs allow to dissect the distinct contributions of telomeric DDR to cells’ fate.

### tASO effects in low-level progerin-expressing and HGPS cells

**Fig. 2:**
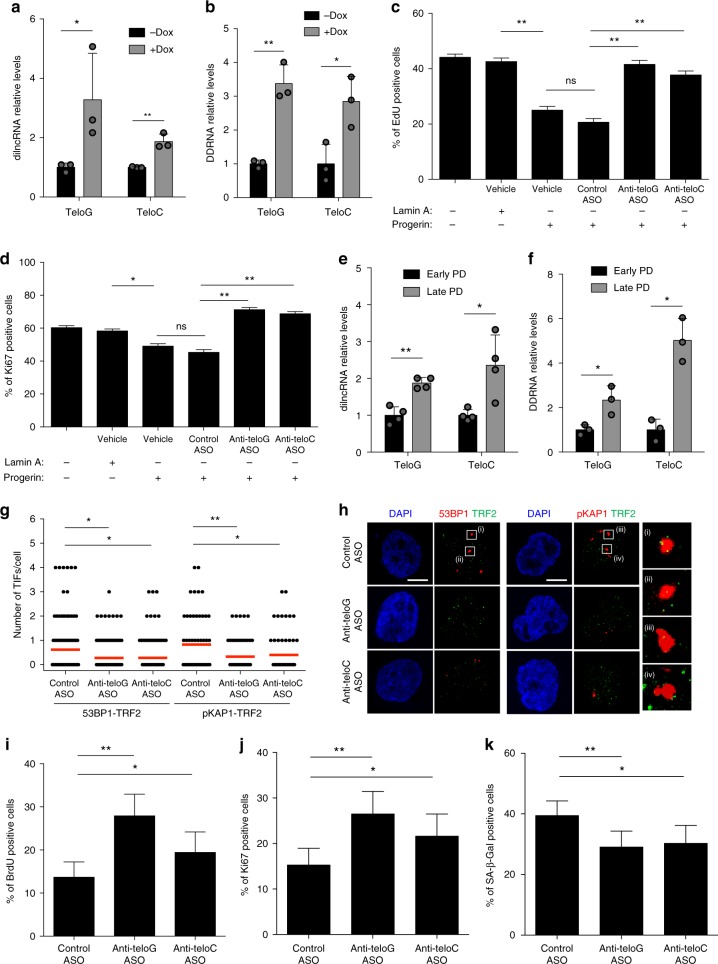
Low levels of progerin expression and lamin A G608G mutation cause telomeric dilncRNAs and DDRNAs accumulation and their inhibition reduces proliferative defects and cellular senescence. **a**, **b** Total cell RNA was isolated from human fibroblasts carrying a doxycycline (Dox)-inducible progerin lentiviral-based system. **a** tdilncRNAs were quantified by strand-specific RT-qPCR. Error bars represent s.d., *n* = 3 independent experiments. *P < 0.05, **P < 0.01; two-tailed Student’s *t* test. **b** tDDRNAs were quantified by miScript PCR amplification of gel-extracted small RNAs (shorter than 40 nucleotides). Error bars represent s.d., *n* = 3 independent experiments. *P < 0.05, **P < 0.01; two-tailed Student’s *t* test. **c**, **d** Lamin A, Progerin-expressing, and control normal dermal fibroblasts (NDF) were transfected with the indicated ASOs. After 9 days cells were pulsed with EdU for 8 h and stained for EdU (**c**) and Ki67 (**d**). Bar graphs show the percentage of EdU and Ki67-positive cells ± 95% confidence interval. *n* = 3 independent experiments. **P < 0.01; Chi-squared test. At least 1000 cells per sample were analyzed. **e**, **f** Total cell RNA was isolated from HGPS patient-deriv**e**d cells at early and late population doubling (PD). **e** tdilncRNAs were quantified as in **a**. Error bars repr**e**sent the s.d. *n* = 4 independent experiments. *P < 0.05, **P < 0.01; two-tailed Student’s *t* test. **f** tDDRNAs were quantified as in **b**. Error bars represent the s.d. *n* = 3 independent experiments. *P < 0.05; two-tailed Student’s *t* test. **g** Late PD HGPS patient fibroblasts were transfected with the indicated ASOs and stained for 53BP1 or pKap1 (red) and TRF2 (green) to quantify TIFs. Co-localization analysis was assessed as in Fig. 1c. *n* = 3 independent experiments. *P < 0.05; one-way ANOVA with multiple-comparison post-hoc corrections. At least 100 cells per sample were analyzed. **h** Representative stack images from quantifications shown in **g**. Scale bars, 10 μm. **i**–**k** HGPS patient fibroblasts from the experiment shown in **g** were pulsed with BrdU for 24 h pr**i**or to fixation and stained for BrdU (**i**), Ki67 (**j**), and SA**-**β-Gal activity (**k**). Bar graphs show the percentage of BrdU, Ki67, and SA-β-Gal**-**positive cells ± 95% confidence interval. *n* = 3 independent experiments. *P < 0.05, **P < 0.01; Chi-squared test. At least 300 cells per sample were analyzed. Source data are provided as a Source Data file

The results described so far were generated in cells constitutively expressing relatively high levels of progerin after retroviral delivery. To study the generation of tncRNAs in cells expressing progerin at the levels observed in HGPS cells, we employed a recently developed doxycycline-inducible lentiviral-based system which allows the tunable expression of progerin^17^. Also in this model we observed an increase in both tdilncRNAs and tDDRNAs upon progerin expression (Fig. 2a, b). Telomeric DDR inhibition by the use of tASOs, but not control ASO, significantly increased cell proliferation rates, as independently evaluated by EdU incorporation and the percentage of Ki67-positive cells (Fig. 2c, d) and decreased the percentage of SA-β-gal-positive cells (Supplementary Fig. 2d, e). These effects were not due to altered progerin expression, as progerin levels remained unaffected by ASO treatments (Supplementary Fig. 2f). Similarly, ASO treatments had no effect on heterochromatin marks, lamin B1 levels, and telomere length (Supplementary Fig. 2f–g and 2h, i, respectively).

We next investigated the role of tncRNAs in primary dermal fibroblasts from HGPS patients^32^. To this end, we compared early and late population doubling (PD) HGPS cells approaching cellular senescence and being ten PD older. Consistent with previous reports^38^, we detected heightened progerin levels in late PD cells compared with early PD cells (Supplementary Fig. 3a, b); this was associated with increased numbers of TIFs, as independently measured by pKap1 and 53BP1 DDR markers (Supplementary Fig. 3c–e) and decreased proliferation rates as measured by BrdU and Ki67 (Supplementary Fig. 3f, g) in late PD cells. The quantification of tdilncRNAs and tDDRNAs revealed higher levels in late PD HGPS cells (Fig. 2e, f), indicating that tncRNA levels are physiologically modulated in HGPS cells as they proliferate in culture and approach premature cellular senescence. To study the role of tncRNAs in these cells, we inhibited their functions by transfecting tASOs in late PD HGPS cells. We observed that both anti-teloG and anti-teloC ASOs, but not control ASO, significantly reduced the number of TIFs (Fig. 2g, h). Moreover, ASO-based inhibition of tdilncRNAs and tDDRNAs led to an increase in BrdU- and Ki67-positive cells (Fig. 2i, j) and reduced the number of SA-β-gal-positive cells (Fig. 2k and Supplementary Fig. 3h) without altering progerin levels (Supplementary Fig. 3i, j), heterochromatin marks (Supplementary Fig. 3k), and TERRA levels (Supplementary Fig. 3l). Importantly, RT-qPCR analysis of mRNA levels revealed that inflammatory cytokines commonly associated with cellular senescence, namely IL-1a, IL-6, and IL-8, were generally reduced upon tASO, compared with control ASO treatment (Supplementary Fig. 3m).

Altogether, these results demonstrate that tdilncRNAs and tDDRNAs play an important role in telomeric DDR activation caused by progerin and that their sequence-specific ASO-mediated inhibition improves the proliferative potential of HGPS cells and reduces their premature entry into cellular senescence.

### tASOs inhibit telomeric DDR in a skin mouse model of HGPS

The skin is one of the first organs to show typical signs of disease in HGPS patients. These include scleroderma-like skin changes, loss of subcutaneous adipose tissue, and alopecia^39^. To test whether telomeric DDR signaling contributes in a relevant way to these HGPS phenotypes in vivo, we employed a conditional HGPS mouse model in which progerin is expressed in the keratin 5 (K5)-positive compartment of the skin^40^. This model recapitulates several features of the HGPS skin phenotypes as described previously^40^.

**Fig. 3:**
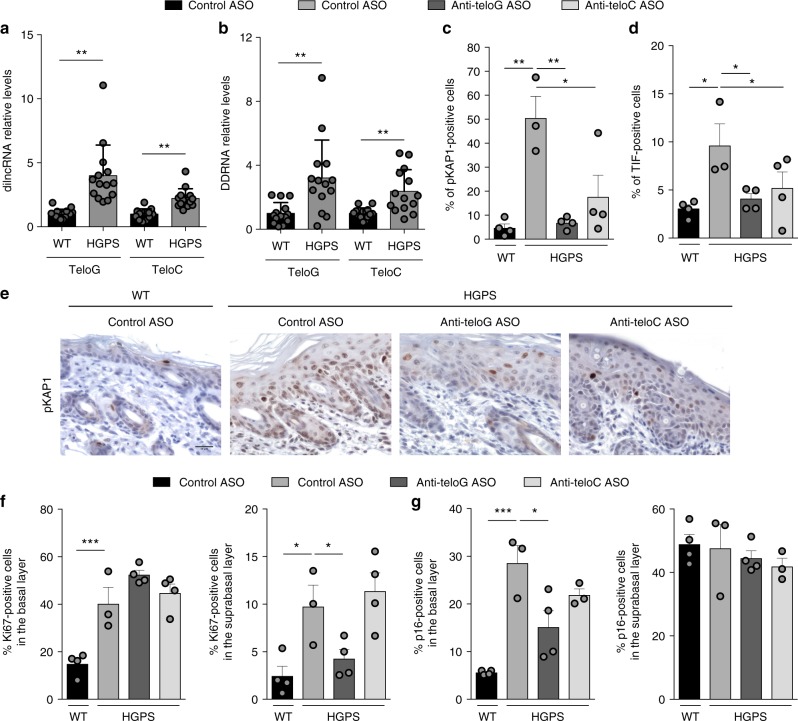
A mouse skin model of HGPS shows increased levels of tncRNAs and their inhibition in vivo reduces DDR activation and cellular senescence. **a**, **b** Total cell RNA was isolated from the skin of wild type (WT) and HGPS mice at post natal days 3 to 8. **a** tdilncRNAs were quantified by strand-specific RT-qPCR. Error bars represent the s.d. *n* = 14 mice per group. **P < 0.01; two-tailed Student’s *t* test. **b** tDDRNAs were quantified by miScript PCR amplification of gel-extracted small RNAs (shorter than 40 nucleotides). Error bars represent the s.d. *n* = 14 mice per group. **P < 0.01; two-tailed Student’s *t* test. **c**–**g** Mice subjected to systemic delivery of the indicated ASOs were sacrificed at post natal day 6 and skin samples were stained for DDR and proliferation markers. Mouse skin sections were immunohistochemically stained for pKAP1 (**c**, **e**) and positive cells were quantified in the epidermis. Scale bar, 50 μm. **d** Mouse skin sections were stained for pKAP1 and TRF1 to quantify telomere dysfunction-induced foci (TIFs) as determined by pKAP1 co-localizing with TRF1 in the basal layer of the skin. A cell was counted as positive if showing at least one TIF. *n* = 3 independent experiments. **P < 0.01; one-way ANOVA with multiple-comparison post-hoc corrections. At least 300 cells per sample were analyzed. Quantification of Ki67 (**f**) and p16 (**g**) positive cells in the supra basal and basal layers of epidermis. Error bars represent the s.d. *n* = 3–4 mice per group. *P < 0.05, **P < 0.01, ***P < 0.001; one-way ANOVA with multiple-comparison post-hoc corrections. At least 300 cells per sample were analyzed. Source data are provided as a Source Data file

When we tested the levels of tncRNAs in skin samples of wild-type (WT) and HGPS mice by RT-qPCR, we observed higher levels of both tdilncRNAs and tDDRNAs in HGPS mice as compared with WT (Fig. 3a, b), indicative of significant telomeric dysfunction in this HGPS model.

We previously reported that systemic treatment with tASOs through intraperitoneal injection effectively inhibits DDR activation in vivo as observed in liver and kidney in an inducible *Trf2* knockout mouse model^25^. To assess the impact of treatment with tASOs in HGPS mice, pregnant mice were systemically injected with anti-teloG, anti-teloC, or control ASOs at embryonic day 17. After birth, new-born mice received additional ASO treatments by intraperitoneal injections every 3 days, starting at post natal day 2. Immunohistochemical analyses of control ASO-treated mouse epidermis showed a higher number of cells that stained positive for the ATM target pKAP1 and 53BP1 compared with similarly treated WT mice (Fig. 3c, e and Supplementary Fig. 4a, d, respectively). Strikingly, such increased levels of markers of DDR activation in HGPS mice were significantly reduced upon tASOs treatment (Fig. 3c, e and Supplementary Fig. 4a, d), with a more robust effect observed in anti-teloG-treated mice. When TIFs were analyzed, we observed increased levels in HGPS mice compared with WT and a significant reduction in HGPS mice treated with tASO but not control ASO (Fig. 3d and Supplementary Fig. 4b) in the absence of detectable telomere lengthening (Supplementary Fig. 4c). Mouse epidermis is made of a basal and suprabasal layer of cells and proliferation is confined to the basal layer, while suprabasal layer is composed of differentiated elements in a quiescent state^41^. We determined the number of proliferating cells in ASO-treated skin by immunohistochemistry against Ki67. In agreement with previous studies in this HGPS mouse model^40^, the overall proliferation of epidermal cells, expressed as the percentage of Ki67-positive cells, was increased, likely due to a perturbed homeostasis, as compared with that of WT mice. Such an increase was mainly contributed by the induction of an aberrant proliferative activity within the differentiative, suprabasal, layers of the epidermis, which under normal conditions are quiescent (Fig. 3f and Supplementary Fig. 4e). Interestingly, anti-teloG treatment induced a significant decrease of the pathological proliferation observed in the suprabasal layer of HGPS skin down to levels observed in WT animals (Fig. 3f and Supplementary Fig. 4e) while the proliferative fraction of the basal layer was not significantly affected. These results indicate that anti-teloG can restore homeostatic proliferation. This effect on keratinocyte proliferation was best observed by combined K5 and Ki67 immunostaining (Supplementary Fig. 4e) which also allowed to exclude a contribution of K5-negative nonepithelial proliferating cells (e.g., immune cells) in the epidermis. Within the same compartments, we also evaluated the number of cells expressing p16, a marker of cellular senescence. In the basal epidermis of HGPS mice, we observed a significantly higher number of p16-positive cells compared with WT animals, which was reduced by anti-teloG treatment—p16 was widely expressed in the suprabasal layer both in WT and HGPS mice and unaffected by treatments (Fig. 3g and Supplementary Fig. 4f). Taken together, these results indicate that in vivo sequence-specific targeting of tncRNAs by tASOs successfully inhibits DDR signaling, limits the pathological induction of p16 in the skin basal layer, and controls aberrant suprabasal layer cells proliferation, reverting key pathological features of this skin-specific HGPS mouse model.

### tASOs improve skin homeostasis in HGPS mice

**Fig. 4:**
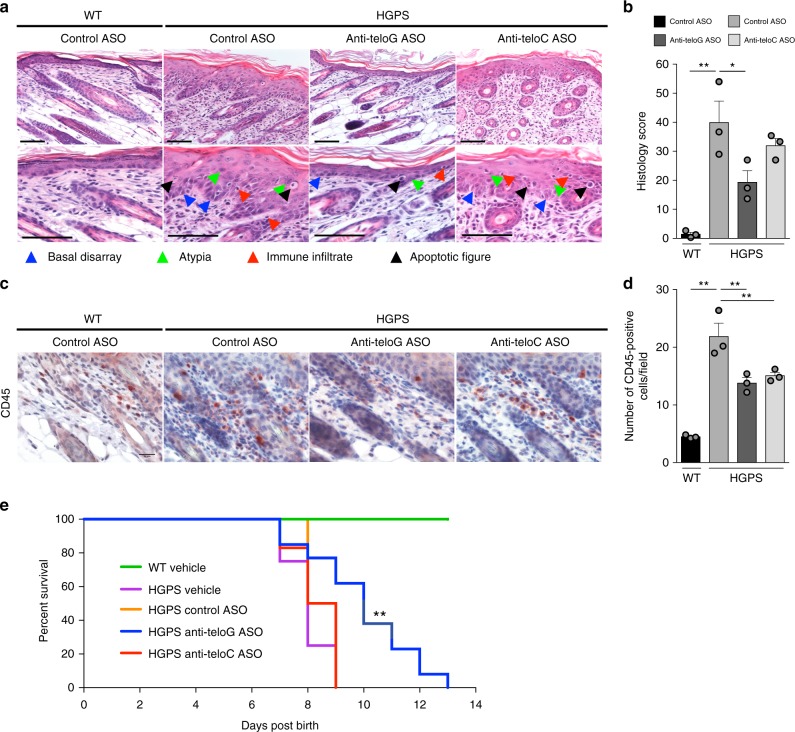
Systemic delivery of tASOs in a mouse skin model of HGPS reduces skin inflammation and degeneration and extends maximal and mean animal survival. **a**–**d** Mice subjected to systemic delivery of the indicated ASOs were sacrificed at post natal day 6 for histopathological characterization. **a** Hematoxylin and eosin stained skin sections of mice treated with the indicated ASOs. Scale bars, 100 μm. **b** Histopathology scores represent the cumulative analysis of the individual parameters shown in Supplementary Fig. 4b–f. Error bars represent the s.d. *P < 0.05, **P < 0.01; one-way ANOVA with multiple-comparison post-hoc corrections. **c** Mouse skin sections were immunohistochemically stained for CD45. Scale bar, 50 μm. **d** Quantifications of images shown in **c**. Error bars represent the s.d. **P < 0.01; one-way ANOVA with multiple-comparison post-hoc corrections. Color scales are assigned as for **b**. **e** Kaplan–Meier curve of wild type (WT) mice treated with vehicle (*n* = 16) and progerin-expressing mice treated with vehicle (*n* = 8), control (*n* = 4), anti-teloG (*n* = 13), or anti-teloC ASOs (*n* = 7). **P < 0.01. Kaplan–Meier survival analysis. Source data are provided as a Source Data file

To study the impact of reduced DDR activation by tASOs in the skin of HGPS mice, we performed a thorough histopathological characterization of the skin epidermal and dermal layers. Progerin expression in this HGPS mouse model has previously been reported to induce severe skin abnormalities, impairing homeostasis, and development^40,42^. Indeed, in HPGS mice we observed epidermal hyperplasia with hyperparakeratosis, basal layer disarray (i.e., alteration in the shape, orientation, and cell-to-cell adhesion of basal cells), nuclear pleiomorphism and atypias, immune cells infiltrate, along with increased apoptotic/necrotic figures, as illustrated in Fig. 4a. These morphological modifications in the epidermis were associated with alterations of the dermal layer thickness ratio, increased dermal inflammatory infiltration and stromal fibrotic remodeling, hyperplasia, and irregular maturation of the sebocytes in sebaceous glands (Supplementary Fig. 5a–h).

To determine the impact of tASO treatment on these pathological features, we graded the severity of these skin alterations according to a combined semiquantitative histopathological score (see “Methods” section) on haematoxylin and eosin (H&E)-stained sections from ASO-treated mice. Overall, we observed that tASO, but not control ASO, treatments reverted, to different extents, the histomorphological alterations of HPGS samples. Specifically, the degree of epidermal hyperplasia, architectural basal layer disarray, the extent of keratinocyte nuclear atypias, and the dermal stromal remodeling were significantly decreased by anti-teloG treatment (Fig. 4a, b and Supplementary Fig. 5a–e). The observed increase in epidermal and dermal thickness was also reduced by both tASOs (Supplementary Fig. 5g, h). Expression of progerin arrests skin development at post natal day 4 with a significant multilayered appearance compared with the WT:^42^ we observed that treatment with tASOs—anti-teloG more than anti-teloC—allowed a less multilayered appearance, as observed by the analysis of epidermal hyperplasia and thickness, and by a somehow more preserved pool of cells expressing Keratin 15 (K15), a marker of epidermal stem cells, in the basal layer (Supplementary Fig. 6a, b). Importantly, tASO, but not control ASO, treatments reduced the number of morphologically evident apoptotic/necrotic figures in H&E sections, which was associated with an overall reduction of keratinocyte apoptosis as independently assessed by TUNEL assays (Fig. 4a and Supplementary Figs. 5f, 6c). Finally, in agreement with an improved progression of skin development, an overall assessment of the macroscopic phenotype of mice indicated a significantly more preserved hair growth in the anti-teloG-treated compared with control-treated HGPS mice from day 8 and onwards (Supplementary Fig. 6d).

We next evaluated the impact of tASO treatments on skin inflammation. By H&E staining we observed that HGPS mice showed heightened levels of immune cells infiltrate in both the dermal and epidermal compartments compared with WT animals (Fig. 4a). Quantification of the number of infiltrating cells expressing CD45, a pan-leukocyte marker, demonstrated their prominent increase in the dermis of HGPS mice, compared with WT animals, which was significantly reduced upon treatment with both tASOs (Fig. 4c, d). Both In situ and RT-qPCR analyses of mRNA expression of inflammatory factors in the skin of WT and HGPS ASO-treated mice indicated an overall increase of inflammatory cytokines in the skin of HGPS mice, with IL-1a transcripts mainly associated with the suprabasal epidermis, particularly at the upper granular layer, while IL-6 and IL-8 transcripts mostly localized within the dermis (Supplementary Fig. 7). Consistently, HGPS mice showed increased cytokine expression levels compared with WT mice and tASO treatments variably affected their expression, demonstrating a statistically-significant decrease for IL-8 within the dermal layer (Supplementary Fig. 7h), and for IL-1a and IL-8 when whole skin RNA was analyzed by RT-qPCR (Supplementary Fig. 7c, i).

Taken together, these results indicate that ASO-mediated inhibition of telomeric DDR signaling in HGPS mice skin has beneficial effects as indicated by improved tissue homeostasis and reduced inflammation—noteworthy and differently from our observations in cultured cells, anti-teloC ASO had a generally milder effect, consistent with its poorer in vivo biodistribution as observed in the skin samples analyzed (Supplementary Fig. 8a, b).

### Anti-teloG ASO administration extends lifespan of HGPS mice

Given the positive histological outcomes induced by telomeric ASO treatment and most evidently by anti-teloG, we investigated whether the systemic administration of ASOs could impact the survival of HPGS mice. To this end, similarly to the treatment schedule described above, pregnant mice were injected with ASOs until death. Importantly, systemic ASO treatments were well tolerated by both control and HGPS mice, with no remarkable weight loss, indicating the absence of toxicities associated with their administration (Supplementary Fig. 8c). As previously reported, these transgenic HGPS mice have a dramatically shorter lifespan compared with WT animals^40,42^, with a median survival of 8 days. Strikingly, treatment with anti-teloG ASO significantly improved the survival of HPGS mice: maximum lifespan increased by 44% and median lifespan by 24%, as compared with control ASO-treated mice (Fig. 4e). Under the conditions employed, anti-teloC ASO treatment did not significantly alter the lifespan of HGPS mice, in line with reduced bioavailability and consistently less strong effects on DDR and histopathological features and.

In summary, these results demonstrate that sequence-specific targeting of progerin-induced tdilncRNAs and tDDRNAs by ASOs allows telomeric DDR inhibition both in cultured human cells and in vivo, resulting in improved skin homeostasis of progeric mice and in a significant extension of their survival.

## Discussion

Organismal aging is characterized by telomere dysfunction and consequent DDR activation and accumulation of senescent cells^28,31,43–45^. Animal models of telomere dysfunction, such as that caused by telomere shortening upon telomerase inactivation in mice, accelerate several features of physiological aging^46–49^. However, the actual contribution of DDR activation at dysfunctional telomeres to organismal aging remains uncharacterized. This is also due to the fact that until now, no experimental approach allowed telomere-specific DDR inactivation. We have previously shown that tASOs can achieve efficient DDR inhibition at telomeres both in cultured cells and in vivo in mice undergoing telomere dysfunction upon TRF2 genetic loss^25^. However, their use in a physiological and clinically relevant condition associated with telomeric DDR remained untested. We used this experimental approach to determine the contribution of telomere dysfunction and DDR activation in HGPS as a paradigm of progeroid conditions. HGPS is associated with a number of cellular and organismal alterations including and beyond telomere dysfunction^39,50^.

Here we showed the impact of efficient inhibition of DDR signaling emerging from telomeres in different HGPS cells systems and tissues by the use of inhibitory ASOs targeting tdilncRNAs and tDDRNAs, the tncRNAs necessary for full DDR activation^25^. Treatment with telomeric ASOs in three HGPS cell systems rescued the proliferative defects and the entry into cellular senescence without impacting on other features of progeroid cells such as nuclear envelope shape alterations and loss of heterochromatin. These results demonstrate the relevant contribution of telomere dysfunction in the senescent phenotype of HGPS cells.

In the skin of mice expressing progerin we observed DDR activation in keratinocytes and the accumulation of tdilncRNAs and tDDRNAs, supporting the notion that tncRNAs are specific in vivo biomarkers of telomere dysfunction. ASO-mediated inhibition of tncRNAs in the skin of HGPS mice led to a dramatic reduction of DDR activation and to a significant improvement of macroscopic and histopathological features of HPGS skin damage, either intrinsic to keratinocytes, or related to increased inflammatory cell infiltration and stromal remodeling. The effects of tASO treatment proved to be more conspicuous through the administration of anti-teloG, which ultimately led to a significant increase in the lifespan of HGPS mice. Anti-teloC treatment showed an overall milder beneficial effect at the tissue level and did not affect mice survival. Since anti-teloG and anti-teloC ASO were equally effective in telomeric DDR inhibition and rescue of proliferative defects when tested in cultured cells, the observed in vivo differences are likely due to a suboptimal tissue distribution of anti-teloC ASO, as indicated by its reduced detection in the skin of this mouse model.

These results demonstrate that telomere dysfunction is a causative molecular mechanism of HGPS pathogenesis and that controlling DDR at telomeres in a sequence-specific fashion may represent an effective approach to improve the phenotypes of HGPS. Most importantly conceptually, our results in cultured cells and in mice indicate that not DNA damage per se but the consequent DDR activation is responsible for the detrimental effects observed.

It is worth reminding that these conclusions may have impact beyond HGPS. The so-called telomere syndromes are a collection of conditions associated with telomere dysfunction^51^ in which the pathogenetic role of DDR activation consequent to telomere dysfunction is unclear. Until recently, it was not possible to selectively inhibit DDR at telomeres and monitor its effects. Here, we have provided evidence that the use of ASOs against ncRNAs is an efficient way to inhibit DDR signaling at dysfunctional telomeres in a relevant animal model, and we have validated ASOs as a potent therapeutic agent for HGPS and potentially for any other disease caused by telomeric dysfunction.

## Methods

### Cell culture

BJ cells (ATCC) were grown in MEM, supplemented with 10% fetal bovine serum (FBS), 1% l-glutamine, 1% nonessential amino acids, and sodium pyruvate 1 mM. Phoenix amphotropic cells (ATCC) were grown in DMEM, supplemented with 10% FBS, and 1% l-glutamine. HGPS patient-derived human primary fibroblasts were grown in DMEM, supplemented with 20% FBS, and 1% l-glutamine. Informed consent had been obtained for these cells, which were donated to CNR Institute of Molecular Genetics by patient family to be used for research on HGPS. Samples belong to BioLaM biobank at CNR Institute of Molecular Genetics Unit of Bologna located in the Rizzoli Orthopedic Institute, Bologna, Italy. Normal dermal fibroblasts (NDF) harboring pTRIPZ-v5-lamin A or pTRIPZ-v5-progerin^17^ were grown in MEM, supplemented with 15% FBS, and 1% l-glutamine, in the presence of 1 μg ml^−1^ puromycin. For induction of progerin expression, NDFs were cultured in the presence of doxycycline (2 μg ml^−1^) for 4 days. All cells were grown at 37 °C, 5% CO_2_.

### Retroviral transduction

Retrovirus producer Phoenix amphotropic cells were transfected with expression vectors pLPC-lamin A and pLPC-Progerin (Addgene plasmids numbers 69059 and 69061, respectively). Forty eight hours post transfection, the concentrated viral supernatants were collected and used to perform four rounds of infections in BJ human fibroblasts for a period of 2 days. After infection, BJ cells were selected for 2 days in the presence of puromycin at a concentration of 2 μg ml^−1^.

### Animals and treatments

Mice were housed in within a pathogen-free animal facility at the Karolinska Institutet, Huddinge, Sweden, and maintained in a 12-h light/dark cycle, at 20–22 °C, and 50–65% air humidity. Mice were supplied with RM3 pellets (Scanbur, Sweden) and drinking water ad libitum. This study was performed in accordance with the institutional guidelines and regulations. Animal studies were approved by the Stockholm South Ethical review board, Dnr. 35–15. Breedings and genotyping were in accordance with previously described procedures^40,42^. ASOs dissolved in PBS were intraperitoneally (i.p.) injected at a concentration of 15 mg kg^−1^ from embryonic day 17 (i.p. injections of mothers), and once every 3 days after birth, starting from post natal day 2 and until death. For histology and immunohistochemical analysis, ASO-injected mice were sacrificed at post natal day 6. Dorsal skin tissues were collected and either frozen and included in optimal cutting temperature compound or fixed in 4% paraformaldehyde (PFA) and embedded in paraffin.

### RNA isolation

Total RNA from cultured cells was extracted with the Maxwell RSC miRNA Kit (Promega) or with the mirVana miRNA Isolation kit (Life Technologies) for mRNA, DDRNA, and dilncRNA detection, according to the manufacturer’s instructions. Snap frozen skin tissue from mice was homogenized with a TissueLyser II (Qiagen) at 30 Hz for 60 s and total RNA was extracted with the Maxwell RSC miRNA Kit (Promega), according to the manufacturer’s instructions.

### Real-time quantitative PCR

One micrograms of total RNA was reverse transcribed using the SuperScript VILO cDNA Synthesis Kit. A volume corresponding to 5 ng of initial RNA was employed for each real-time PCR reaction using SYBR Green I Master Mix (Roche) on a Roche LightCycler 480 detection system. Each reaction was performed in triplicate. Human and mouse Human ribosomal protein lateral stalk subunit P0 (Rplp0) were used as control transcripts for normalization. TERRA transcripts were quantified using subtelomeric primers described in ref. ^37^. Primers sequences (5−3′ orientation) were:

Rplp0 (mouse and human) Fw: TTCATTGTGGGAGCAGAC

Rplp0 (mouse and human) Rv: CAGCAGTTTCTCCAGAGC

Progerin Fw: ACTGCAGCAGCTCGGGG

Progerin Rv: TCTGGGGGCTCTGGGC

IL-6 Fw (mouse): GAGGATACCACTCCCAACAGACC

IL-6 Rv (mouse): AAGTGCATCATCGTTGTTCATACA

IL-8 Fw (mouse): GTCCTTAACCTAGGCATCTTCG

IL-8 Rv (mouse): TCTGTTGCAGTAAATGGTCTCG

IL-1a Fw (mouse): CTACAGTTCTGCCATTGACC

IL-1a Rv (mouse): TTGAGCGCTCACGAACAGTT

IL-6 Fw (human): CAGCCCTGAGAAAGGAGACAT

IL-6 Rv (human): GGTTCAGGTTGTTTTCTGCCA

IL-8 Fw (human): TTGGCAGCCTTCCTGATTTC

IL-8 Rv (human): TCTTTAGCACTCCTTGGCAAAAC

IL-1a Fw (human): GGTTGAGTTTAAGCCAATCCA

IL-1a Rv (human): TGCTGACCTAGGCTTGATGA

### Real-time quantitative PCR for small RNAs

One micrograms of total RNA was fractionated on a 20% polyacrylamide, 7 M Urea gel, and RNA species shorter than 40 nucleotides were gel extracted. cDNA was synthesized using the miScript II RT kit (Qiagen) with HiSpec buffer. Real-time PCR was performed using the miScript PCR system (Qiagen), according to the manufacturer’s instructions. Each reaction was performed in triplicate. mir17 was used as a control transcript for normalization. Primer sequences (5−3′ orientation):

mir17 CAAAGTGCTTACAGTGCAGGTAG

teloG TAGGGTTAGGGTTAGGGT

teloC CCCTAACCCTAACCCTAA

### Strand-specific real-time quantitative PCR

Detection of tdilncRNAs was performed as previously described^25^. Briefly, RNA samples were treated with DNase I (Thermo Scientific) at 37 °C for 1 h. Next, 1 μg of total RNA was reverse transcribed using the Superscript First Strand cDNA synthesis kit (Invitrogen) with strand-specific primers. qPCR was performed using SYBR Green I Master Mix (Roche). A volume of cDNA corresponding to 8 ng of initial RNA was used. Each reaction was performed in triplicate. Rplp0 was used as a control gene for normalization. Primer sequences (5−3′ orientation):

Rplp0 Fw TTCATTGTGGGAGCAGAC

Rplp0 Rv CAGCAGTTTCTCCAGAGC

teloC Rv CCCTAACCCTAACCCTAA

teloG Rv TTAGGGTTAGGGTTAGGG

telo Fw CGGTTTGTTTGGGTTTGGGTTTGGGTTTGGGTTTGGGTT

telo Rv GGCTTGCCTTACCCTTACCCTTACCC TTACCCTTACCCT

### Transfection

Transfections were carried out with Lipofectamine RNAiMAX (Invitrogen) according to the manufacturer’s instructions.

### ASOs sequences

The ASOs used were locked nucleic acid mixmer oligonucleotides with a fully phosphorothioate backbone (Exiqon). They were used at a final concentration of 20 nM for transfection of cultured cells and 15 mg kg^−1^ for mouse injections. Sequences were as follows (5−3′ orientation):

Control ASO TTATCCGCTCACAATTCCACAT

anti-teloG ASO CCCTAACCCTAACCCTAACCC

anti-teloC ASO GGGTTAGGGTTAGGGTTAGGG

### Telomere restriction fragment (TRF) analysis

Genomic DNA was extracted from flash frozen cell pellets or skin tissues with the Qiagen DNeasy Blood and Tissue kit. gDNA was digested with the AluI and MboI restriction enzymes, and separated on a 0.7% agarose gel using either standard gel electrophoresis (human cell pellets samples), or pulsed-field gel electrophoresis (mouse skin tissue samples). DNA was then transferred to a nitrocellulose membrane, and probed with a P32-labeled TTAGGG repeat probe (pSP73.Sty11; addgene #12401). Probed membranes were exposed to a phosphorimager screen, and subsequently imaged on a Typhoon Imager (GE).

### Immunoblot

Cells were lysed in Laemmli buffer (2% SDS, 10% glycerol, 60 mM Tris HCl pH 6.8). Thirty micrograms of whole cell lysates were resolved by SDS polyacrylamide gel electrophoresis. Proteins were transferred to a nitrocellulose membrane and subsequently blocked in 5% milk in TBST (Tris-Buffered Saline 0.1% Tween). Primary antibodies were incubated overnight at 4 °C and horseradish peroxidase (HRP)-conjugated secondary antibodies were incubated for 1 h at RT. Image acquisition was performed with a ChemiDoc Imager (Bio-Rad). Uncropped and unprocessed scans of all immunoblots are available in the Source Data file.

### Immunofluorescence for cultured cells

Cells were fixed with 4% PFA solution. After incubation with blocking solution, cells were stained with primary antibody for 1 h at RT, washed, and incubated with secondary antibodies for 45 min at RT. Nuclei were stained with DAPI (1 μg ml^−1^). Samples were mounted in mowiol solution (Calbiochem). For BrdU incorporation, cells were labeled with 10 μg ml^−1^ BrdU (Sigma) for 8–24 h and incorporation was evaluated by immunofluorescence after DNA denaturation. NDFs were labeled with 10 μM EdU for 8 h and EdU was detected using the Click-IT EdU (Alexa 488) protocol (Thermo Fisher Scientific).

### Senescence-associated-β-galactosidase assay (SA-β-Gal)

Cells were washed in PBS, fixed for 10 min in 4% PFA, washed, and incubated at 37 °C (in the absence of carbon dioxide) with fresh SA-β-Gal stain solution (pH 6.0): Potassium ferricyanide 5 mM, Potassium ferrocyanide 5 mM, Sodium dihydrogen phosphate 0.4 M, Sodium hydrogen phosphate 92 mM, Sodium chloride 150 mM, Magnesium dichloride 2 mM, and 5-bromo-4-chloro-3-indolyl-β-D-galactopyranoside 1 mg ml^−1^. Staining was evident in 2–4 h and maximal in 12–16 h.

### Imaging

Immunofluorescence images were acquired using a wide field Olympus Biosystems Microscope BX61 or a GE Healthcare DeltaVision Elite high-resolution fluorescence microscope. For co-localization between DDR markers and telomeres, software-based image deconvolution of DeltaVision acquisitions was performed in order to generate optical sections at different levels along the *z* axis of the cell. Co-localization was assessed by ImageJ software with a customized ImageJ macro to allow 3D stack analysis. Two points were considered co-localizing if their respective intensities were higher than the threshold of their channels and if five pixels or more overlapped between both channels within the same section of the stack. Olympus wide field microscopes were used for the remaining imaging experiments (BrdU, Ki67, and DDR markers). Comparative immunofluorescence analyses were performed in parallel with identical acquisition parameters. Tissue immunofluorescence images were acquired using a Nikon A1R and A1+ imaging systems (Nikon Corporation, Japan). Images were analyzed using NIS elements (Nikon Corporation, Japan). Immunohistochemical images of p16 were acquired using either a Zeiss Axioplan 2 microscope (Carl Zeiss AG, Germany) coupled to the Zeiss Axiocam MRm camera or a Zeiss Axioscope A1 (Carl Zeiss AG, Germany) equipped with a Zeiss Axiocam 503 digital camera. Images were analyzed with the Image-Pro Insight 9.1 software and with the Zen 2 image analysis software.

### Circularity image analysis

Processing of each DeltaVision Elite high-resolution image consisted of a consecutive series of algorithms implemented as plugins in the freely available software ImageJ (http://imagej.nih.gov/ij/). Briefly, the image is deconvoluted into separate color channels and subsequently, the lamin A/C channel is extracted and used for pixel intensity-based threshold segmentation. For circularity quantification, the outlines of segmented nuclei are determined using edge detection algorithms based on differential brightness cutoffs. Circularity indexes range from 1.0 (representing a perfect circle) to 0 (representing a straight line).

### In situ hybridization

IL-1a, IL-6, IL-8, and K15 transcripts were detected using RNAscope (Advanced Cell Diagnostic) in accordance with the manufacturer’s protocol with only a few modifications: target retrieval was performed for 20 min and Protease Plus was applied on sections for 30 min. A minimum of 3 dots per cell was set to be counted as a positive cell. RNAscope assay was followed by addition of anti-keratin 5 antibody in order to identify the epidermal basal layer. Secondary antibody was added and sections were counterstained with DAPI (1:1000, ThermoFisher Scientific) prior to mounting.

### Chromogenic in situ hybridization

In situ hybridization was performed on 4-µm-thick formalin-fixed paraffin-embedded skin tissue sections using custom biotinylated probes (Exiqon) with the following sequences (5−3′ orientation):

To detect anti-teloC ASO: CCCTAACCCTAACCCTAACCC/3Bio/

To detect anti-teloG ASO: GGGTTAGGGTTAGGGTTAGGG/3Bio/

Following deparaffinization and endogenous peroxidase quenching with 3% H_2_O_2_, pre treatment was performed at 98–104 °C for 15 min followed by protease treatment at 40 °C for 15 min. The biotinylated probes were used at a final concentration of 5 µM and hybridized at 40 °C for 2 h. ISH signal was detected by incubating sections for 30 min at room temperature with HRP-conjugated streptavidin and DAB (3,3′-Diaminobenzidine) substrate-chromogen. The slides were counterstained with haematoxylin.

### Histopathological examination

Dorsal skin samples were fixed in 4% PFA at 4 °C overnight. Following fixation, the samples were transferred to 70% ethanol, dehydrated, and embedded in paraffin. Paraffin-embedded tissues were cut into 4-μm sections and routinely stained with haematoxylin and eosin (H&E) for histopathological analysis.

For the histopathological evaluation of skin damage, a semiquantitative scoring system was applied, which included the following variables: epidermal hyperplasia, basal layer disarray, keratinocyte nuclear atypia, keratinocyte apoptotic/necrotic figures, and dermal stromal remodeling. All the variables were morphologically evaluated and scored according to the degree of severity (0, absent; 1, mild; 2, moderate; 3 severe) and extent (1, focal; 2, multifocal; 3, diffuse). An overall morphological skin damage score was calculated for each sample as the product of the degree of severity and the extent.

### Tissue immunohistochemistry

Paraffin-embedded tissues were cut into 4-μm sections. Tissue sections were rehydrated and subjected to heat-induced epitope retrieval by incubation in sodium citrate buffer (10 mM, pH 6.0 or pH 9) in a water bath. Endogenous peroxidase activity was blocked using a solution of 2.5% hydrogen peroxide in methanol, followed by specimen blocking with 1.5% normalized goat, rabbit, or rat serum. Primary antibodies were applied to sections followed by overnight incubation at 4 °C. Sections were incubated with either biotinylated-goat anti-rabbit secondary antibody (1:800, Invitrogen), followed by the label antibody (ABC Elite, Vector Laboratories), or with horseradish peroxidase-conjugated donkey anti-rabbit secondary antibody (1:500, Nove by Life Technologies). Enzymatic activity was revealed using 3–3′-diaminobenzidine chromogenic substrate (Dako Cytomation). Mayer’s hemotoxylin (Histolab) was used as counsterstain. Tissue sections were mounted with mounting medium for light microscopy (Pertex, Histolab).

### Automated quantification of nuclear DDR markers

For automated quantification of nuclear DDR markers, whole sample scans were obtained using a Leica Aperio ScanScope CS slide scanner (Leica Biosystems) and the Aperio Image Scope software (version 12.3.2.8013). From the whole scans of each IHC-stained section, five nonoverlapping high-power microscopic fields were extracted and the epidermis was manually segmented. Nuclear segmentation and assessment of nuclear positivity was then automatically determined by the Nuclear Hub Image Analysis package and the result was expressed as a percentage. Positive nuclei were also automatically scored according to the staining intensity (low, intermediate, and high).

### Antibodies

Anti-lamin A/C (Santa Cruz, sc6215, 1:1000 and Cell Signaling Technology, 2032T, 1:1000); anti-BrdU (Becton Dickinson, 347580, 1:20), anti-Ki67 (Abcam, ab16667, 1:50); anti-TRF2 (Millipore, 05–521, 1:200); anti-Tubulin (Sigma-Aldrich, T5168, 1:2000); anti-HP1α (Sigma-Aldrich, H2164, 1:2000); anti-H3K9me3 (Millipore, 05–1242, 1:2000); anti-lamin B1 (Abcam, ab16048, 1:5000); anti-p16 (Santa Cruz Biotechnology, sc-1207, 1:800); anti-Keratin5 (BioSite, PRB-160P, 1:500 and Abcam, ab52635, 1:100); anti-phospho KAP-1 (S824) (Bethyl Laboratories, A300–767A, 1:200); anti-53BP1 (Novus Biologicals, NB100–304, 1:1000), Anti-CD45, (Abcam, ab10558, 1:500).

### Statistical analysis

Results are shown as mean ± standard error of the mean (s.e.m.) or standard deviation (s.d.) or as percentages ± 95% confidence interval as indicated. *P* value was calculated by the indicated statistical tests, using Prism software. In figure legends, *n* indicates the number of independent experiments. Survival distributions of the different treatment groups were plotted using the Kaplan–Meier estimator and statistical analysis was performed using log-rank (Mantel–Cox) test.

### Reporting summary

Further information on research design is available in the Nature Research Reporting Summary linked to this article.

## Supplementary information

Supplementary Information

Reporting Summary

Source Data

## Data Availability

The authors state that all data generated during this study are included in the article, its supplementary information file, and the Source Data file, and are available from the corresponding author upon reasonable request.
