# Supplementary material for: Inhibition of DNA damage response at telomeres improves the detrimental phenotypes of Hutchinson–Gilford Progeria Syndrome
Source: Nat Commun. 2019 Nov 18;10:4990. doi: 10.1038/s41467-019-13018-3 (PMC6861280; doi:10.1038/s41467-019-13018-3)
Supplement: Supplementary file 1 — Supplementary Information [file 41467_2019_13018_MOESM1_ESM.pdf]

Supplementary Information

Inhibition of DNA damage response at telomeres improves the detrimental phenotypes of Hutchinson-Gilford Progeria Syndrome

Aguado J et al.,

Supplementary Figure 1

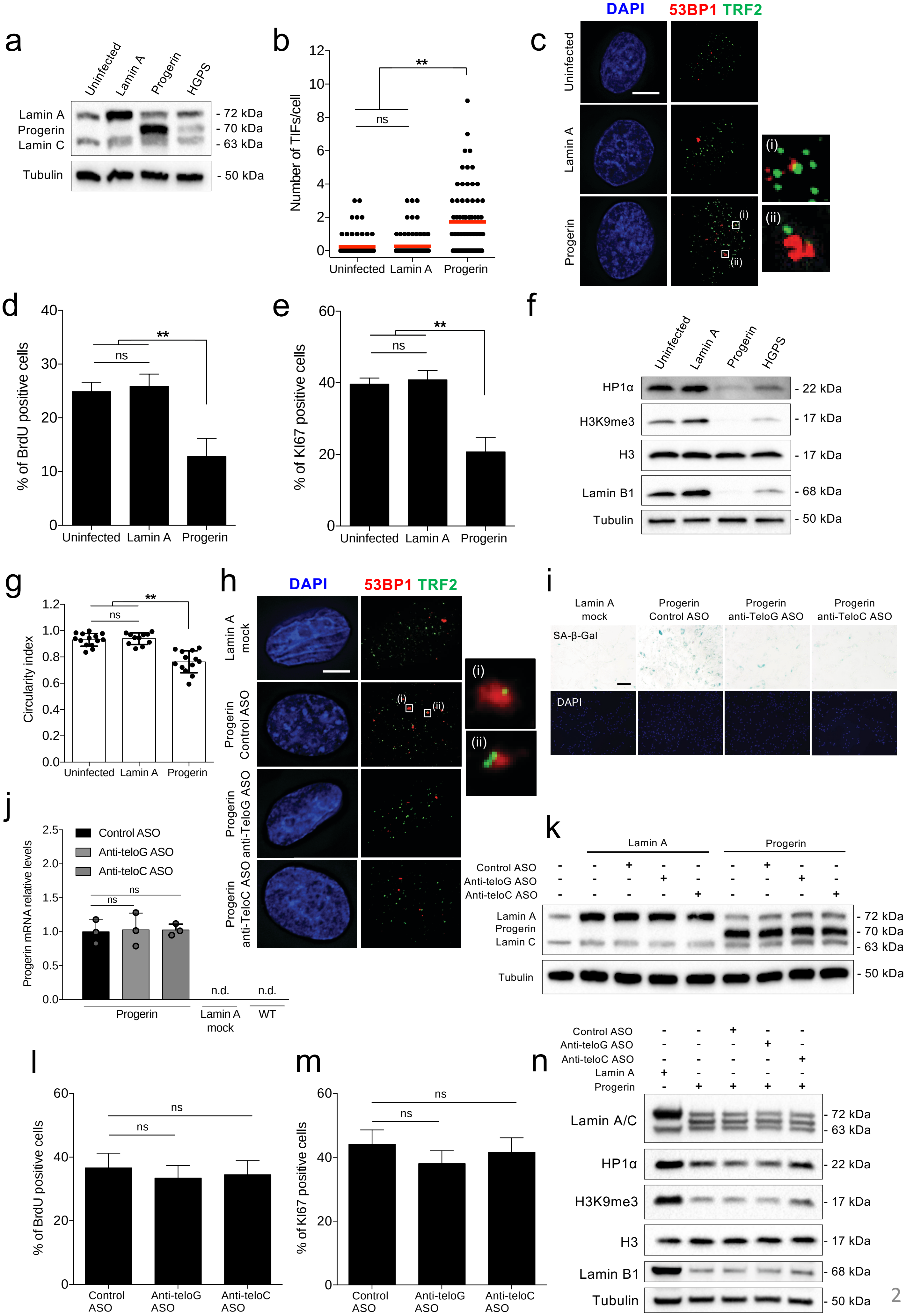

Supplementary Figure 1. (a) Western blot analysis of lamin A and progerin of whole cell extracts from non-infected control human fibroblasts, lamin A or progerin retrovirally-transduced human fibroblasts and HGPS patient fibroblasts. Tubulin was used as loading control and patient fibroblasts as positive control for progerin expression. **(b)** Human fibroblasts were transduced with a retroviral vector expressing either lamin A or progerin. Uninfected and retrovirally-transduced fixed cells were stained for 53BP1 and TRF2 to quantify telomere dysfunction-induced foci (TIFs) as determined by 53BP1 co-localizing with TRF2. n=3 independent experiments. **P<0.01; One-way ANOVA with multiple-comparison Tukey's post-hoc corrections. **(c)** Representative deconvolved stack images from quantifications shown in **b**. Scale bar, 10 μm. **(d,e)** Cells from experiments shown in panel **b** were pulsed with BrdU for 8 hours and stained for BrdU **(d)** and Ki67 **(e)**. Bar graphs show the percentage of positive cells ± 95% confidence interval. n=3 independent experiments. **P<0.01; Chi-squared test. **(f)** Western blot analysis of H3K9me3, HP1α and Lamin B1 of cells from experiments shown in panel **a**. Tubulin was used as loading control. **(g)** Cells from experiments shown in panel **b** were stained for lamin A/C. Circularity quantification of stainings was assessed with a value of 1.0 indicating a perfect circle. As the value approaches 0, it indicates an increasingly elongated shape. Each point in the scatter plot shows the circularity index of a nucleus. Error bars represent the s.d. **P<0.01; One-way ANOVA with multiple-comparison Tukey's post-hoc corrections. **(h)** Representative deconvolved stack images from quantifications shown in Figure 1c. Scale bar, 10 μm. **(i)** Representative images of senescence-associated β-galactosidase (SA-β-Gal) assays shown in Fig. 1f. Scale bar, 100 μm. **(j)** Total RNA from retrovirally-transduced lamin A and progerin-expressing human fibroblasts transfected with the indicated ASOs was used for RT–qPCR to determine progerin mRNA levels. Human ribosomal protein lateral stalk subunit P0 (Rplp0) mRNA was used as normaliser. n=3 independent experiments. One-way ANOVA with multiple-comparison Tukey's post-hoc corrections. **(k)** Western blot analysis for lamin A/C and progerin expression in lamin A and progerin-expressing human fibroblasts transfected with the indicated ASOs. Tubulin was used as loading control. **(l,m)** Lamin A-expressing human fibroblasts transfected with the indicated ASOs were pulsed with BrdU for 8 hours and stained for BrdU **(l)** and Ki67 **(m)**. Bar graphs show the percentage of BrdU and Ki67-positive cells ± 95% confidence interval. **(n)** Western blot analysis of whole cell extracts from human fibroblasts transfected with the indicated ASOs shows the loss of H3K9me3, HP1α and lamin B1 upon progerin expression and unaltered levels upon ASO treatment.

Supplementary Figure 2

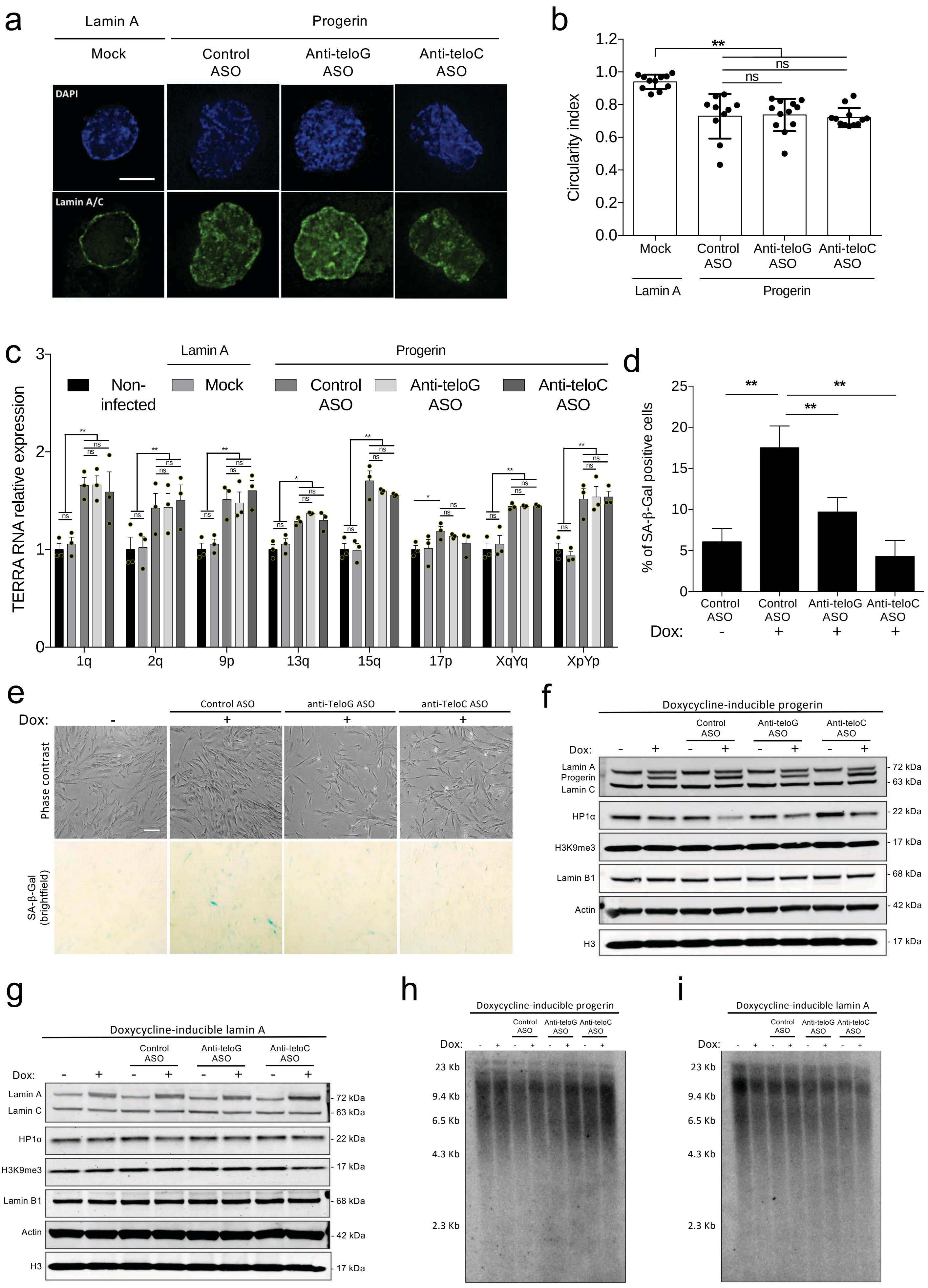

Supplementary Figure 2. (a) Representative deconvolved stack images of lamin A and progerin-expressing human fibroblasts transfected with the indicated ASOs and stained for Lamin A/C. Scale bar, 10 μm. **(b)** Quantification of stainings shown in **a**. Each point in the scatter plot shows the circularity index of a nucleus. **P<0.01; ns: not significant; one-way ANOVA with multiple-comparison Tukey's post-hoc corrections. **(c)** Total RNA from cells in panel **c** were analysed by RT-qPCR for TERRA levels by amplification of different subtelomeric 5' sequences from different telomeres upon normalization on Rplp0 mRNA and non-infected control human fibroblasts. Error bars represent s.d.; *P<0.05, **P<0.01, ns: not significant; n=3 independent experiments. One-way ANOVA with multiple-comparison post-hoc corrections. **(d)** Control and progerin-expressing normal dermal fibroblasts (NDFs) were transfected with the indicated ASOs and stained for senescence-associated β-galactosidase (SA-β-Gal) activity. Bar graphs show the percentage of positive cells ± 95% confidence interval. n=3 independent experiments. **P<0.01; Chi-squared test. **(e)** Representative images of SA-β-Gal experiments shown in panel **d**. Scale bar, 100 μm. **(f,g)** Western blot analyses of lamin A/C, progerin, heterochromatin marks and laminB1 expression in lamin A and progerin-expressing NDFs transfected with the indicated ASOs. Actin was used as loading control. **(h,i)** Telomere restriction fragment (TRF) assay was used to assess telomere length in control, lamin A and progerin-expressing NDFs transfected with the indicated ASOs.

Supplementary Figure 3

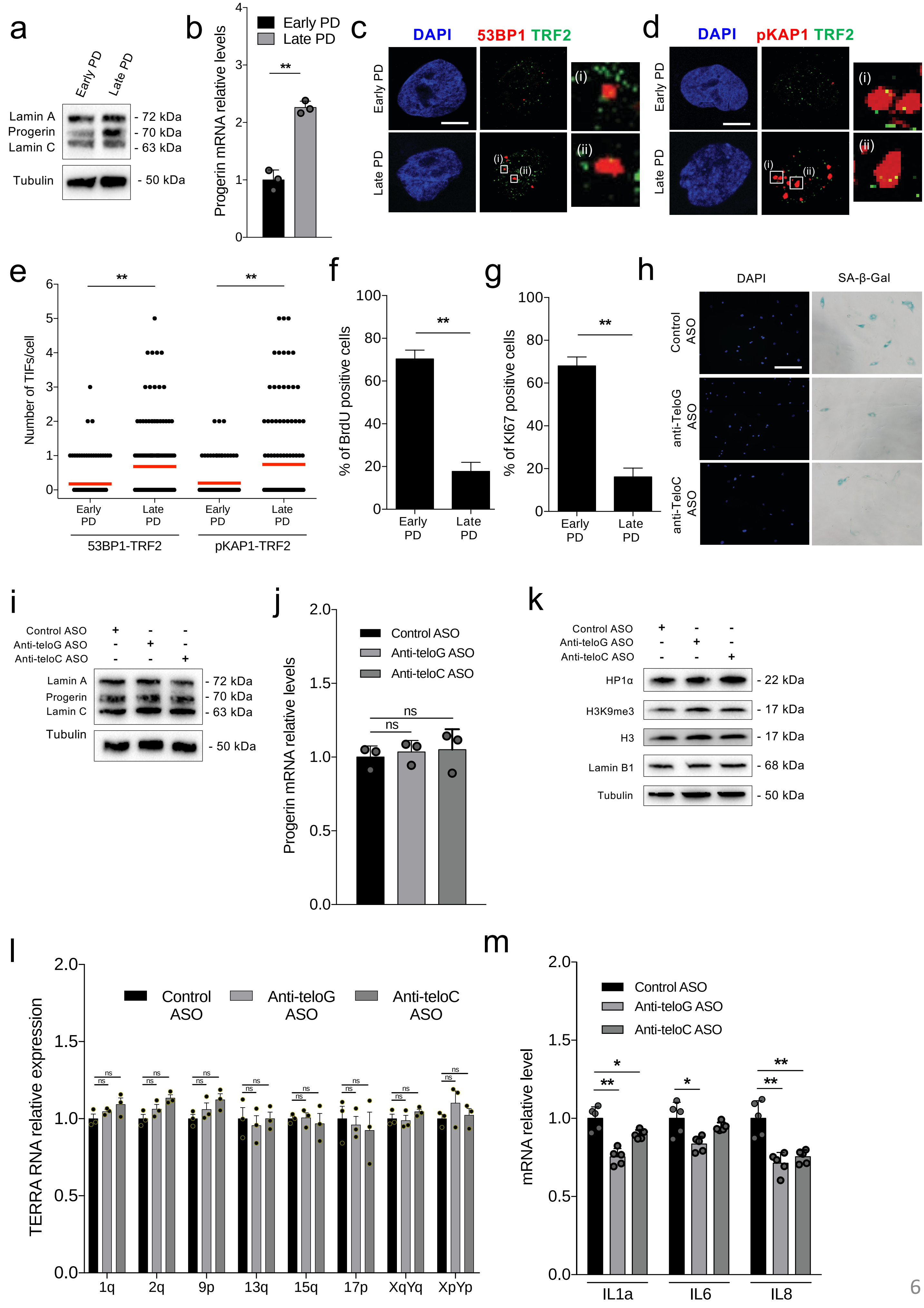

Supplementary Figure 3. (a) Western blot analysis of whole cell extracts from fibroblasts of HGPS patients at early and late population doubling (PD) was performed to assess progerin protein levels. Tubulin was used as loading control **(b)** Total RNA from cells in panel **a** was used for RT–qPCR to detect progerin mRNA levels. Human Rplp0 mRNA was used as normaliser. n=3 independent experiments. Error bars represent the s.d. **P<0.01; two-tailed Student's t-test. **(c-e)** HGPS patient fibroblasts at early and late population doubling (PD) were stained for 53BP1 and TRF2 **(c)** or pKAP1 and TRF2 **(d)** to quantify telomere dysfunction-induced foci (TIFs) as determined by 53BP1 or pKAP1 co-localizing with TRF2 **(e)**. n=3 independent experiments. **P<0.01; two-tailed Student's t-test. At least 100 cells per sample were analysed. Scale bars, 10 μm. **(f,g)** Early and late PD HGPS patient fibroblasts were pulsed with BrdU for 24 hours and stained for BrdU **(f)** and Ki67 **(g)**. Bar graphs show the percentage of BrdU and Ki67 positive cells ± 95% confidence interval. n=3 independent experiments. **P<0.01; Chi-squared test. **(h)** Representative images of the senescence-associated β-galactosidase (SA-β-Gal) experiments shown in Fig. 2k. Scale bar, 100 μm. **(i)** Western blot analysis of whole cell extracts from fibroblasts of HGPS patients at late PD transfected with the indicated ASOs was used to assess progerin levels upon ASO treatment. Tubulin was used as loading control**. (j)** Total RNA from cells in panel **i** was used for RT–qPCR to detect progerin mRNA levels and normalized to Rplp0 mRNA and compared to Control ASO. Error bars represent s.d. ns: not significant. n=3 independent experiments. One-way ANOVA with multiple-comparison Tukey's post-hoc corrections. **(k)** Western blot analyses of whole cell extracts from cells in panel **i** was performed to assess the levels of H3K9me3, HP1α and lamin B1 upon ASO treatment. Tubulin was used as loading control. **(l)** Total RNA from cells in panel **i** were analysed by RT-qPCR and levels of TERRA with different subtelomeric sequences from different telomeres were measured and normalized to Rplp0 mRNA and compared to Control ASO. Error bars represent s.d.; ns: not significant. n=3 independent experiments. One-way ANOVA with multiple-comparison Tukey's post-hoc corrections. **(m)** Total RNA from cells in panel **i** were analysed by RT-qPCR analysis and the levels of IL1a, IL6 and IL8 were measured and normalized to Rplp0 mRNA and compared to Control ASO. Error bars represent s.d.; *P<0.05, **P<0.01; n=5 independent experiments. One-way ANOVA with multiple-comparison Tukey's post-hoc corrections.

Supplementary Figure 4

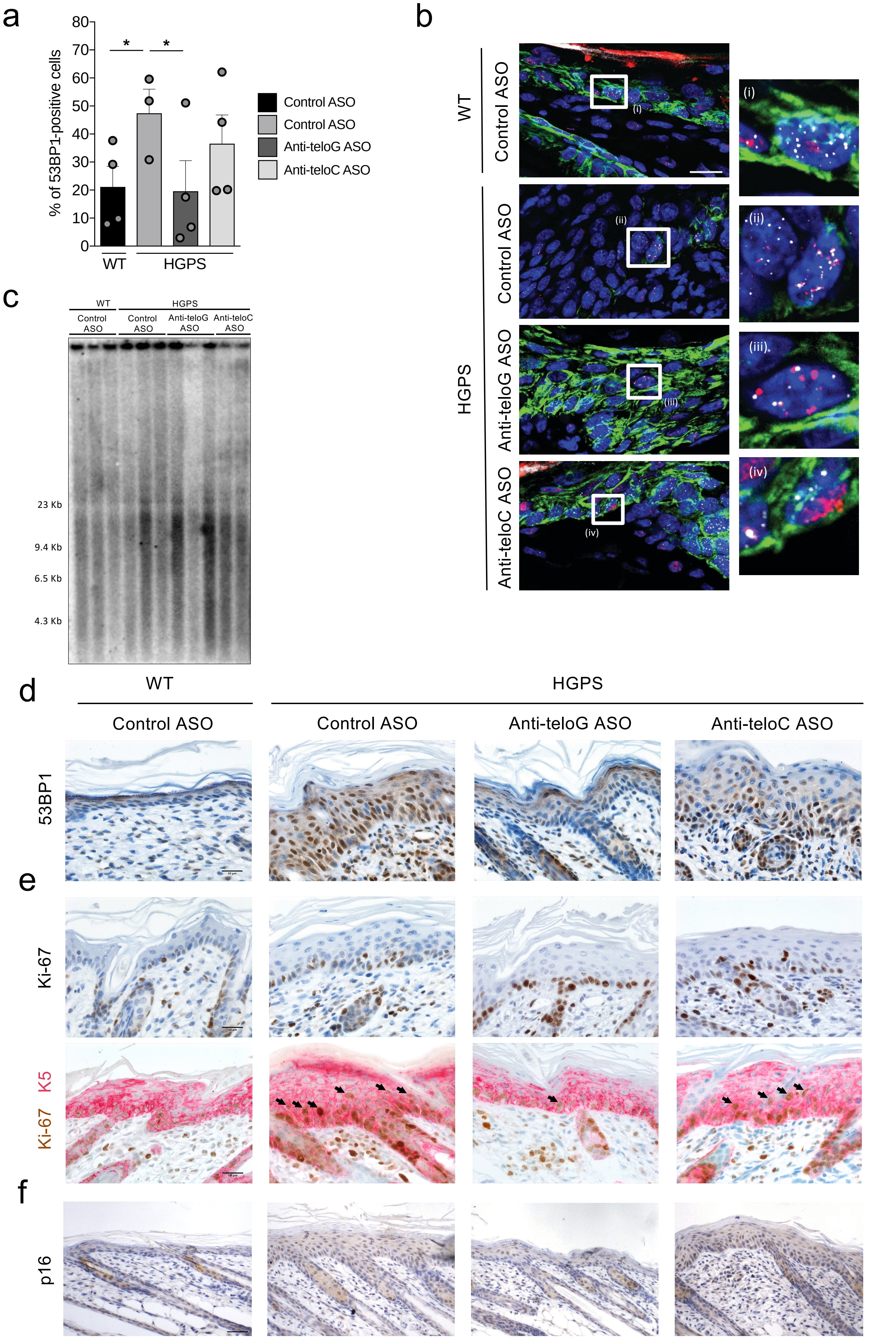

Supplementary Figure 4. Wild type (WT) control and HGPS mice injected intraperitoneally with 15 mg kg^{-1} of the indicated ASOs were sacrificed at post-natal day 6 to stain for proliferation and senescence markers. **(a)** Mouse skin sections were immunohistochemically stained for 53BP1 and positive cells were quantified in the epidermis. Scale bars, 50 μm. **(b)** Representative images from quantifications shown in **Fig 3d**. Colour channels: blue (DAPI), white (TRF1), green (K5) and red (53BP1). Scale bar, 10 μm. **(c)** Telomere restriction fragment (TRF) assay was used to assess telomere length in WT and HGPS mouse skin treated with the indicated ASOs. **(d-f)** Representative images of mouse skin sections stained for 53BP1 **(d)**, Ki-67 and K5 **(e)** and p16 **(f)** quantified in panel a and Figs. 3f and 3g, respectively. Scale bars, 50 μm.

Supplementary Figure 5

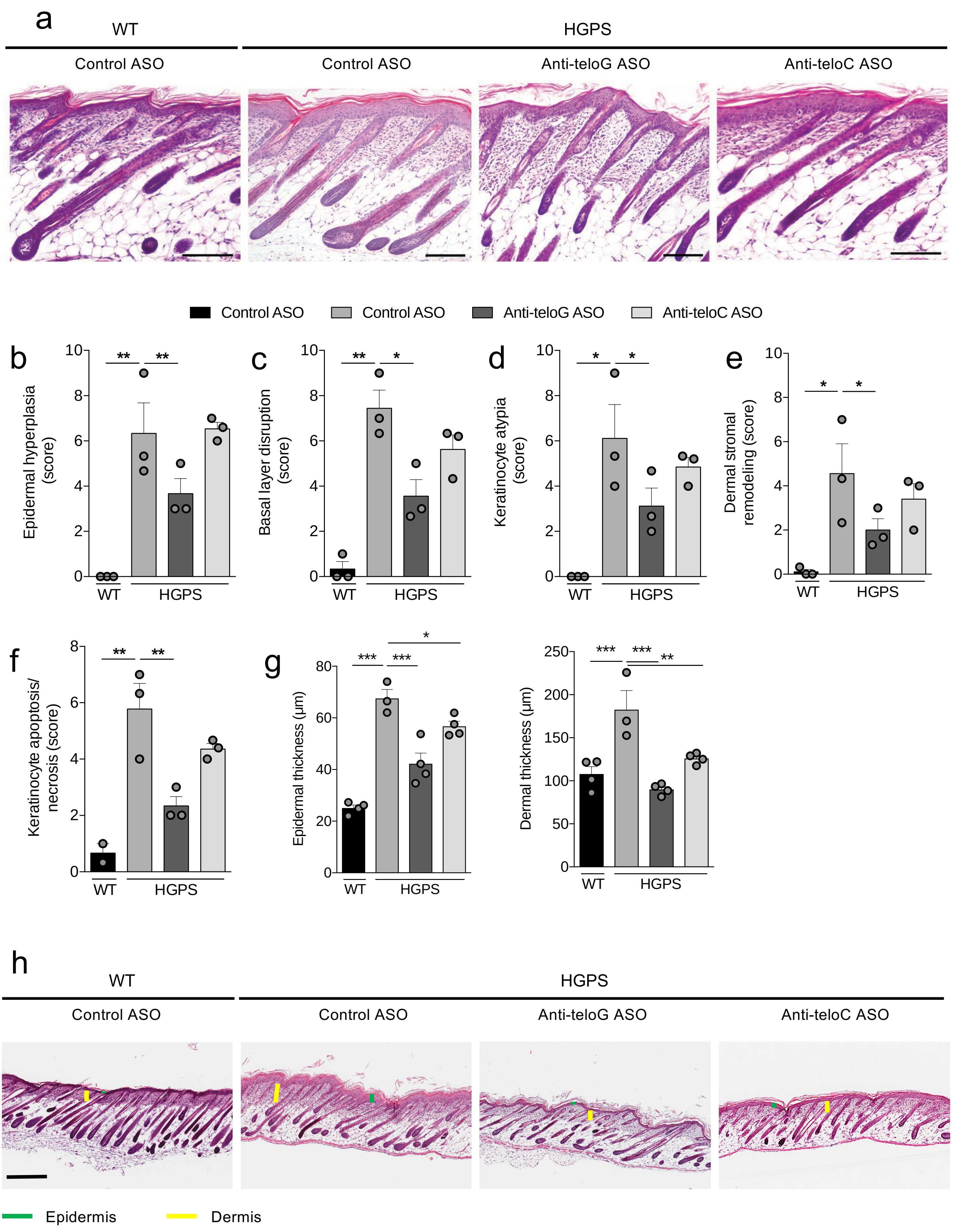

Supplementary Figure 5. (a-h) Wild type (WT) control or HGPS mice were subjected to systemic delivery of the indicated ASOs and sacrificed at post-natal day 6 for analyses. **(a)** Hematoxylin and eosin stained skin sections of mice treated with the indicated ASOs. Scale bars 100 μm **(b-f)** Quantification of individual histological parameters that result in Figure 4b. Error bars represent the s.d. n=3 mice per group. *P<0.05, **P<0.01; one-way ANOVA with multiple-comparison Tukey's post-hoc corrections. **(g)** Quantification of the epidermal and dermal thickness. Error bars represent the s.d. n=3-4 mice per group. *P<0.05, **P<0.01, ***P<0.001; one-way ANOVA with multiple-comparison Tukey's post-hoc corrections. **(h)** Representative images showing epidermal and dermal thickness. Scale bar, 400 μm.

Supplementary Figure 6

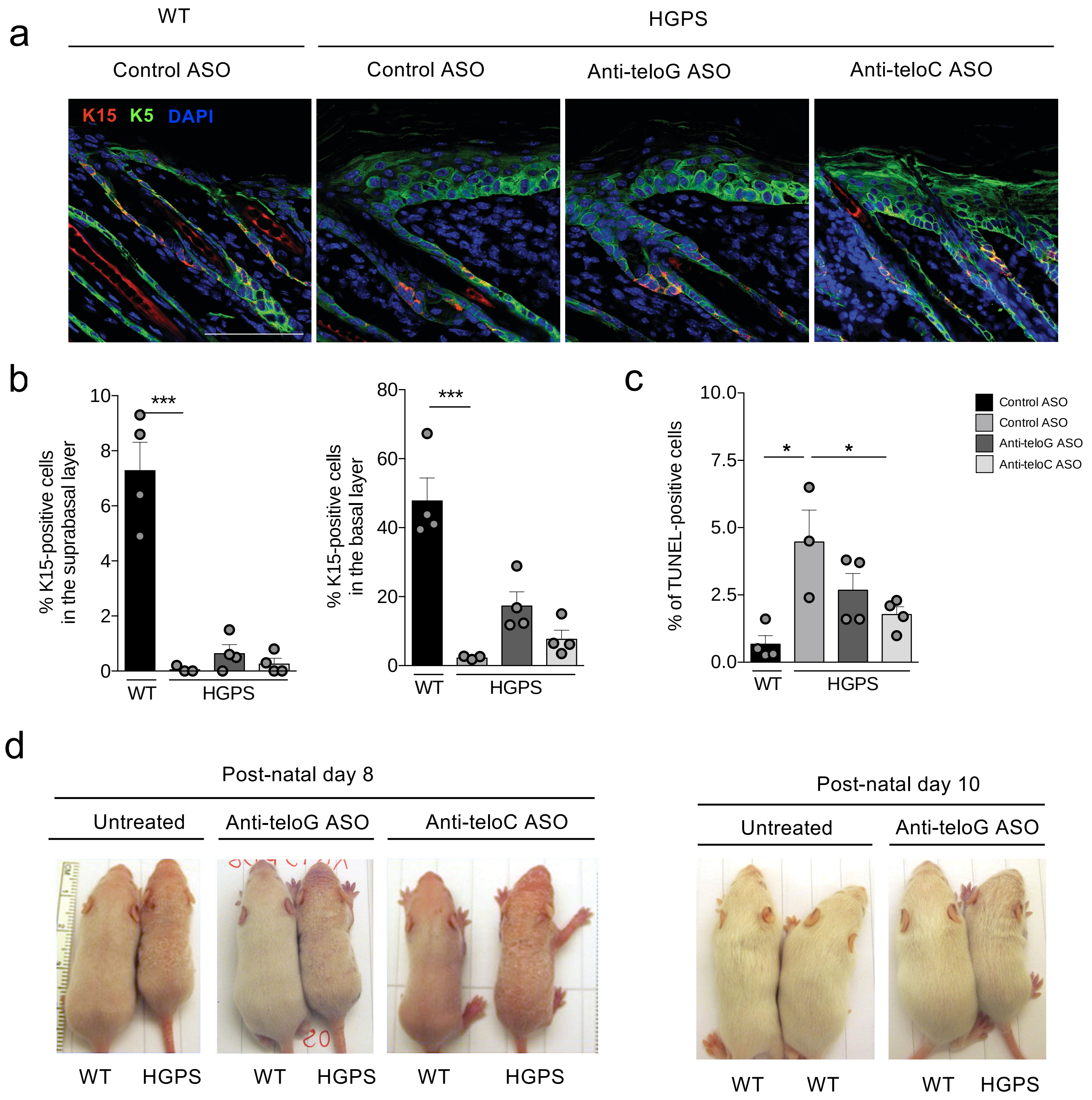

Supplementary Figure 6. Wild type (WT) control or HGPS mice subjected to systemic delivery of the indicated ASOs were sacrificed at post-natal day 6 for stainings. **(a)** Representative images of the RNAscope *in situ* hybridization for K15. Scale bar, 100 μm. **(b)** Quantifications of **a** in the suprabasal and basal layers. Error bars represent the s.d. n=3-4 mice per group. ***P<0.001; one-way ANOVA with multiple-comparison Newman-Keuls post-hoc corrections. **(c)** Quantifications of TUNEL staining in skin sections from mice treated with the indicated ASOs. Error bars represent the s.d. n=3-4 mice. *P<0.05; one-way ANOVA with Tukey's multiple-comparison post-hoc corrections. **(d)** Representative images of mice treated with the indicated ASOs.

Supplementary Figure 7

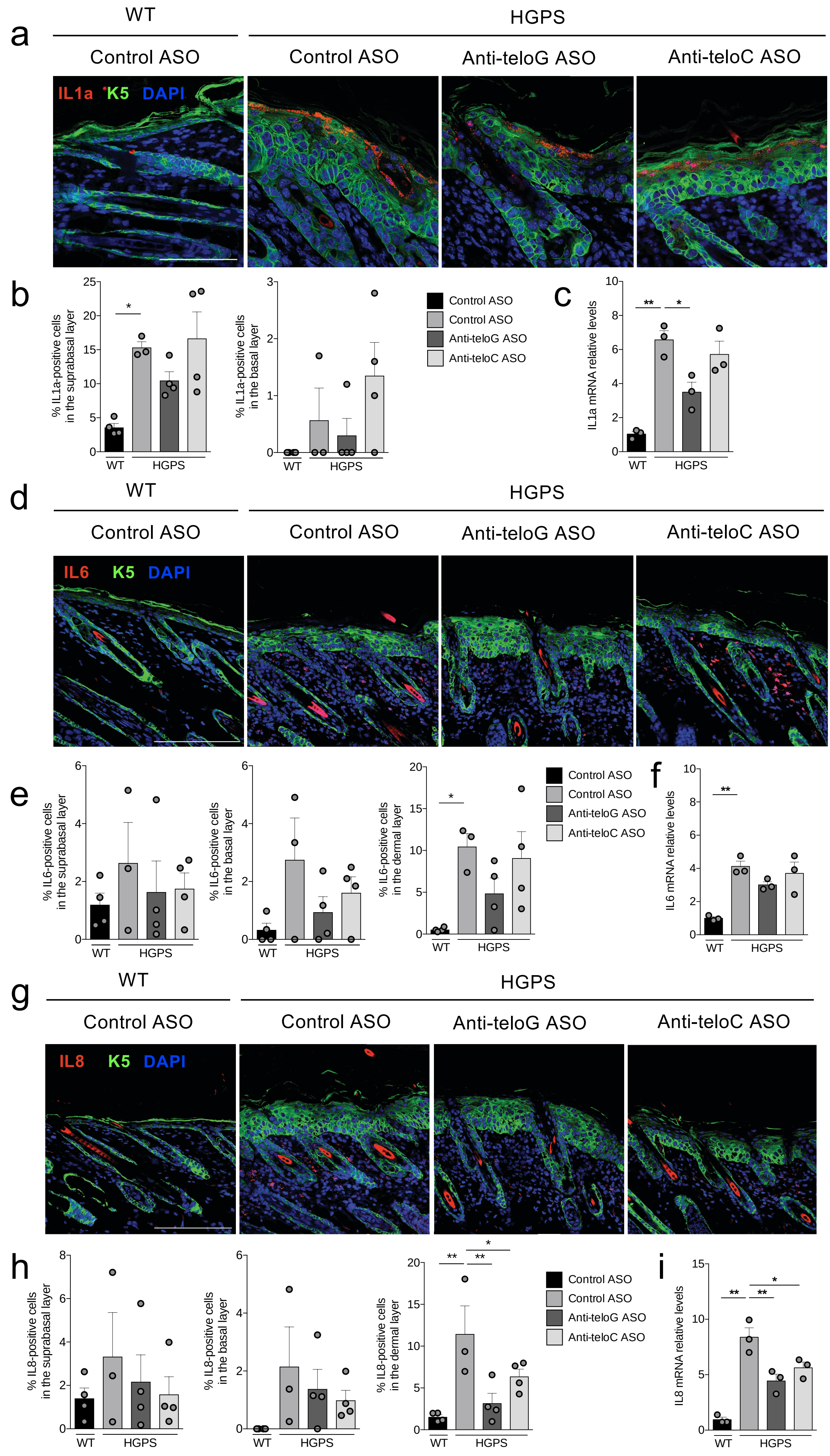

Supplementary Figure 7. Wild type (WT) control or HGPS mice subjected to systemic delivery of the indicated ASOs were sacrificed at post-natal day 6 for stainings. **(a, d, g)** Representative images of the RNAscope *in situ* hybridization for IL1a, IL6 and IL8. Scale bars, 100 μm. **(b, e, h)** Quantifications of **a, d, g** in the suprabasal, basal and dermal layers. Error bars represent the s.d. n=3-4 mice per group. *P<0.05, **P<0.01; one-way ANOVA with multiple-comparison Newman-Keuls post-hoc corrections. **(c, f, i)** Total RNA from whole skin of mice treated with the indicated ASOs was used to quantify the mRNA expression levels of IL1a (c), IL6 (f) and IL8 (i) and normalized to Rplp0 mRNA and compared WT control. Error bars represent s.d.; *P<0.05, **P<0.01; n=3 independent experiments; one-way ANOVA with multiple-comparison Tukey's post-hoc corrections.

Supplementary Figure 8

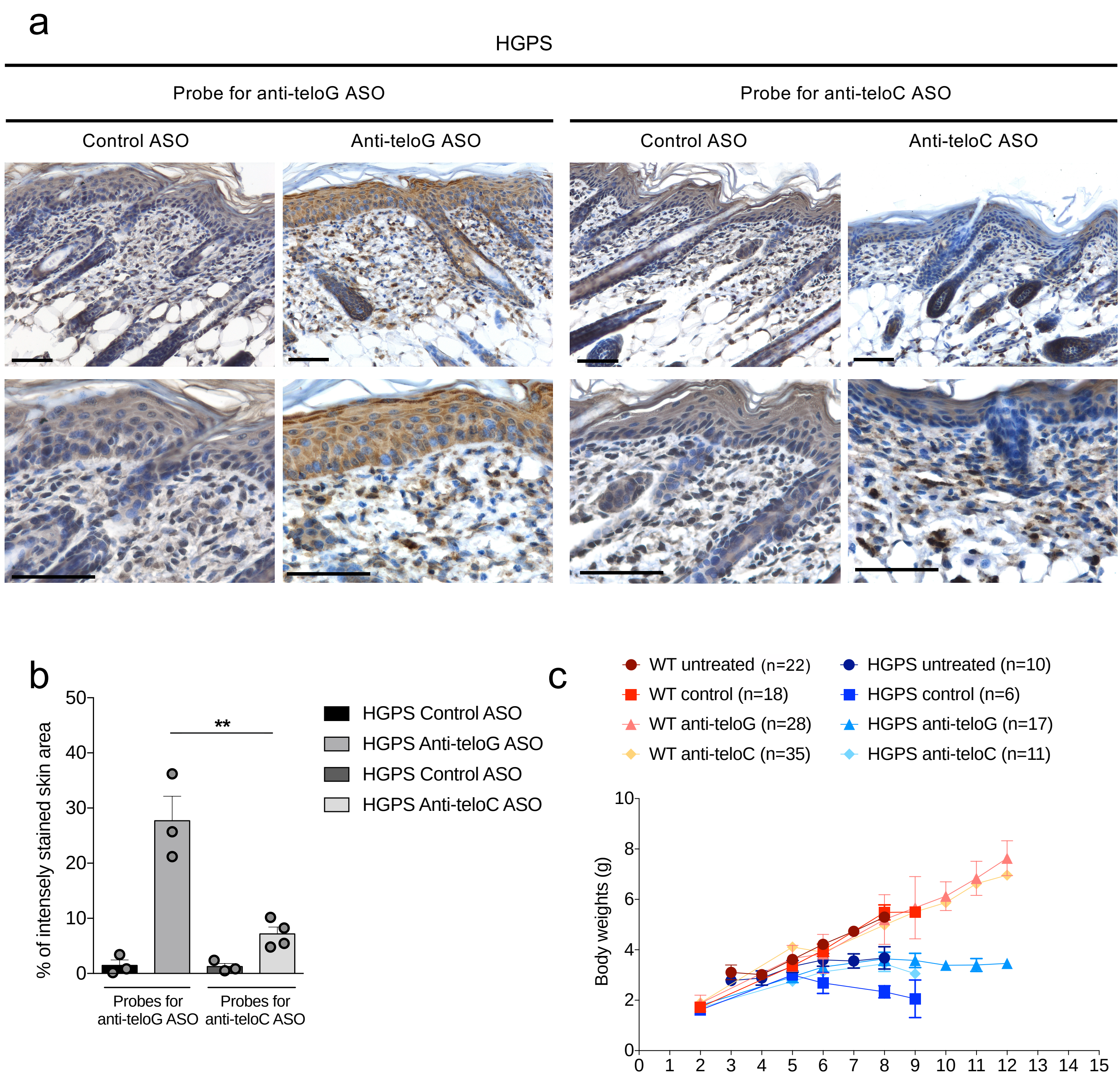

Supplementary Figure 8. (a) HGPS mice subjected to systemic delivery of the indicated ASOs were sacrificed at post-natal day 6 for staining with the indicated probe. Scale bars, 100 μm. **(b)** Quantifications of **a**. Bar graphs show the percentage of intensely stained skin area with the indicated probes in sections from HGPS mice treated with the indicated ASOs. Error bars represent the s.d. n=3-4 mice per group. **P<0.01; one-way ANOVA with multiple-comparison Tukey's post-hoc corrections. **(c)** Wild type (WT) or HGPS mice were subjected to systemic delivery of the indicated ASOs and their body weight was measured at the indicated time points.
