# Supplementary material for: Inhibition of DNA damage response at telomeres improves the detrimental phenotypes of Hutchinson–Gilford Progeria Syndrome
Source: Nat Commun. 2019 Nov 18;10:4990. doi: 10.1038/s41467-019-13018-3 (PMC6861280; doi:10.1038/s41467-019-13018-3)
Supplement: Supplementary file 2 — Reporting Summary [file 41467_2019_13018_MOESM2_ESM.pdf]

nature research

Corresponding author(s): Fabrizio d'Adda di Fagagna

Last updated by author(s): Aug 7, 2019

Reporting Summary

Nature Research wishes to improve the reproducibility of the work that we publish. This form provides structure for consistency and transparency in reporting. For further information on Nature Research policies, see Authors & Referees and the Editorial Policy Checklist.

Statistics

For all statistical analyses, confirm that the following items are present in the figure legend, table legend, main text, or Methods section.

n/a	Confirmed	
☐	☒	The exact sample size (*n*) for each experimental group/condition, given as a discrete number and unit of measurement
☐	☒	A statement on whether measurements were taken from distinct samples or whether the same sample was measured repeatedly
☐	☒	The statistical test(s) used AND whether they are one- or two-sided<br>*Only common tests should be described solely by name; describe more complex techniques in the Methods section.*
☒	☐	A description of all covariates tested
☐	☒	A description of any assumptions or corrections, such as tests of normality and adjustment for multiple comparisons
☐	☒	A full description of the statistical parameters including central tendency (e.g. means) or other basic estimates (e.g. regression coefficient) AND variation (e.g. standard deviation) or associated estimates of uncertainty (e.g. confidence intervals)
☒	☐	For null hypothesis testing, the test statistic (e.g. *F*, *t*, *r*) with confidence intervals, effect sizes, degrees of freedom and *P* value noted<br>*Give P values as exact values whenever suitable.*
☒	☐	For Bayesian analysis, information on the choice of priors and Markov chain Monte Carlo settings
☒	☐	For hierarchical and complex designs, identification of the appropriate level for tests and full reporting of outcomes
☒	☐	Estimates of effect sizes (e.g. Cohen's *d*, Pearson's *r*), indicating how they were calculated

Our web collection on statistics for biologists contains articles on many of the points above.

Software and code

Policy information about availability of computer code

Data collection	WB image collection: Image J (version 1.32j)<br>Data collection: Microsoft Office Excel 2016
Data analysis	Data visualization and analysis : GraphPad Prism (version 5.03)<br>Image analysis: ImageJ (Version: 2.0.0-rc-68/1.52h)<br>Image analysis: Image-Pro Insight 9.1<br>Image analysis: Aperio Image Scope software (version 12.3.2.8013)<br>Data visualization and analysis: Microsoft Office Excel 2016<br>WB image visualization: Image J (version 1.32j)

For manuscripts utilizing custom algorithms or software that are central to the research but not yet described in published literature, software must be made available to editors/reviewers. We strongly encourage code deposition in a community repository (e.g. GitHub). See the Nature Research guidelines for submitting code & software for further information.

Data

Policy information about availability of data

All manuscripts must include a data availability statement. This statement should provide the following information, where applicable:

- Accession codes, unique identifiers, or web links for publicly available datasets
- A list of figures that have associated raw data
- A description of any restrictions on data availability

The authors state that all data generated during this study are included in the article, its supplementary information file, and the Source Data file, and are available from the corresponding author upon reasonable request.

Field-specific reporting

Please select the one below that is the best fit for your research. If you are not sure, read the appropriate sections before making your selection.

☒ Life sciences ☐ Behavioural & social sciences ☐ Ecological, evolutionary & environmental sciences

For a reference copy of the document with all sections, see nature.com/documents/nr-reporting-summary-flat.pdf

Life sciences study design

All studies must disclose on these points even when the disclosure is negative.

Sample size	No statistical analysis methods were used to predetermine sample size estimates. Sample size was determined to be adequate based on the magnitude and consistency of measurable differences between groups.
Data exclusions	No data excluded
Replication	Each experiment was repeated at least three times as described in Figure legends.
Randomization	Mice were assigned according to their genotype. Litter mates and sex and age-matched animals were used whenever possible. All other parameters are random.
Blinding	N/A

Reporting for specific materials, systems and methods

We require information from authors about some types of materials, experimental systems and methods used in many studies. Here, indicate whether each material, system or method listed is relevant to your study. If you are not sure if a list item applies to your research, read the appropriate section before selecting a response.

Materials & experimental systems

n/a	Involved in the study	
☐	☒	Antibodies
☐	☒	Eukaryotic cell lines
☒	☐	Palaeontology
☐	☒	Animals and other organisms
☒	☐	Human research participants
☒	☐	Clinical data

Methods

n/a	Involved in the study	
☒	☐	ChIP-seq
☒	☐	Flow cytometry
☒	☐	MRI-based neuroimaging

Antibodies

Antibodies used	As stated in the Methods section, the following antibodies were used: Anti-Lamin A/C (Santa Cruz, sc6215, 1:1000 and Cell Signaling Technology, 2032T, 1:1000); anti-BrdU (Becton Dickinson, 347580, 1:20), anti-Ki67 (Abcam, ab16667, 1:50); anti-TRF2 (Millipore, 05-521, 1:200); anti-Tubulin (Sigma-Aldrich, T5168, 1:2000); anti-HP1α (Sigma-Aldrich, H2164, 1:2000); anti-H3K9me3 (Millipore, 05-1242, 1:2000); anti-lamin B1 (Abcam, ab16048, 1:5000); anti-p16 (Santa Cruz Biotechnology, sc-1207, 1:800); anti-Keratin5 (BioSite, PRB-160P, 1:500 and Abcam, ab52635, 1:100); anti-phospho KAP-1 (S824) (Bethyl Laboratories, A300-767A, 1:200); anti-53BP1 (Novus Biologicals, NB100-304, 1:1000), Anti-CD45, (Abcam, ab10558, 1:500).
Validation	All the antibodies used in this study were validated by the manufacturers for specific detection of the antigen, human reactivity and western-blot applications.

Eukaryotic cell lines

Policy information about cell lines

Cell line source(s)	-HGPS patient-derived human primary fibroblasts, a gift from Giovanna Lattanzi (Istituto di Genetica Molecolare, Consiglio Nazionale delle Ricerche, Bologna, Italy)<br>-BJ cells, purchased from ATCC (CRL-2522)<br>-Normal primary dermal fibroblasts, a gift from Dr Bruno Reversade (Institute of Medical Biology, A*STAR, Singapore)
Authentication	None of the cell lines used were authenticated.
Mycoplasma contamination	All cell lines involved in this study were tested negative for mycoplasma contamination.

Commonly misidentified lines (See ICLAC register)	N/A

Animals and other organisms

Policy information about studies involving animals; ARRIVE guidelines recommended for reporting animal research

Laboratory animals	Heterozygous tetop-LAG608G or tetop-LAwt (Sagelius et al., 2008) mice were intercrossed with heterozygous K5-tTA mice (Diamond et al., 2000), and offspring were genotyped.
Wild animals	The study did not involve wild animals.
Field-collected samples	The study did not involve samples collected from the field.
Ethics oversight	As stated in the Methods section, mice were housed in within a pathogen-free animal facility at the Karolinska Institutet, Huddinge, Sweden. This study was performed in accordance with the institutional guidelines and regulations. Animal studies were approved by the Stockholm South Ethical review board, Dnr. 35-15.

Note that full information on the approval of the study protocol must also be provided in the manuscript.
